# Comorbidity Patterns in Patients Newly Diagnosed With Colorectal Cancer: Network-Based Study

**DOI:** 10.2196/41999

**Published:** 2023-09-05

**Authors:** Hang Qiu, Liya Wang, Li Zhou, Xiaodong Wang

**Affiliations:** 1 Big Data Research Center University of Electronic Science and Technology of China Chengdu China; 2 School of Computer Science and Engineering University of Electronic Science and Technology of China Chengdu China; 3 Health Information Center of Sichuan Province Chengdu China; 4 Department of Gastrointestinal Surgery West China Hospital Sichuan University Chengdu China

**Keywords:** colorectal cancer, comorbidity patterns, prevalence, health status disparities, network analysis, routinely collected health data

## Abstract

**Background:**

Patients with colorectal cancer (CRC) often present with multiple comorbidities, and many of these can affect treatment and survival. However, previous comorbidity studies primarily focused on diseases in commonly used comorbidity indices. The comorbid status of CRC patients with respect to the entire spectrum of chronic diseases has not yet been investigated.

**Objective:**

This study aimed to systematically analyze all chronic diagnoses and diseases co-occurring, using a network-based approach and large-scale administrative health data, and provide a complete picture of the comorbidity pattern in patients newly diagnosed with CRC from southwest China.

**Methods:**

In this retrospective observational study, the hospital discharge records of 678 hospitals from 2015 to 2020 in Sichuan Province, China were used to identify new CRC cases in 2020 and their history of diseases. We examined all chronic diagnoses using ICD-10 (International Classification of Diseases, 10th Revision) codes at 3 digits and focused on chronic diseases with >1% prevalence in at least one subgroup (1-sided test, *P*<.025), which resulted in a total of 66 chronic diseases. Phenotypic comorbidity networks were constructed across all CRC patients and different subgroups by sex, age (18-59, 60-69, 70-79, and ≥80 years), area (urban and rural), and cancer site (colon and rectum), with comorbidity as a node and linkages representing significant correlations between multiple comorbidities.

**Results:**

A total of 29,610 new CRC cases occurred in Sichuan, China in 2020. The mean patient age at diagnosis was 65.6 (SD 12.9) years, and 75.5% (22,369/29,610) had at least one comorbidity. The most prevalent comorbidities were hypertension (8581/29,610, 29.0%; 95% CI 28.5%-29.5%), hyperplasia of the prostate (3816/17,426, 21.9%; 95% CI 21.3%-22.5%), and chronic obstructive pulmonary disease (COPD; 4199/29,610, 14.2%; 95% CI 13.8%-14.6%). The prevalence of single comorbidities was different in each subgroup in most cases. Comorbidities were closely associated, with disorders of lipoprotein metabolism and hyperplasia of the prostate mediating correlations between other comorbidities. Males and females shared 58.3% (141/242) of disease pairs, whereas male-female disparities occurred primarily in diseases coexisting with COPD, cerebrovascular diseases, atherosclerosis, heart failure, or renal failure among males and with osteoporosis or gonarthrosis among females. Urban patients generally had more comorbidities with higher prevalence and more complex disease coexistence relationships, whereas rural patients were more likely to have co-existing severe diseases, such as heart failure comorbid with the sequelae of cerebrovascular disease or COPD.

**Conclusions:**

Male-female and urban-rural disparities in the prevalence of single comorbidities and their complex coexistence relationships in new CRC cases were not due to simple coincidence. The results reflect clinical practice in CRC patients and emphasize the importance of measuring comorbidity patterns in terms of individual and coexisting diseases in order to better understand comorbidity patterns.

## Introduction

Colorectal cancer (CRC) is the third most common malignancy and the second most deadly cancer globally, with 1.93 million new cases and 0.93 million deaths estimated in 2020 [1]. China has become one of the countries with the highest incidences of CRC cases and CRC-related deaths worldwide, as a consequence of lifestyle changes and accelerated aging of the population [2]. In 2020, newly diagnosed CRC cases in China accounted for 28.8% of new global cases and CRC-related deaths in China accounted for 30.6% of all CRC-related deaths worldwide [3].

Patients with CRC commonly present at an older age and have a relatively high proportion of other chronic diseases (ie, comorbidities). In a retrospective study in Korea, 49.6% of colon cancer patients had ≥3 comorbidities [4]. In an observational cohort study in the United States, 40% of CRC patients had 1 to 3 comorbidities and 19% had ≥4 comorbidities [5]. In addition, evidence is increasing that the coexistence of various chronic diseases has a substantial effect on the treatment, outcomes, and survival of CRC patients [6-9]. In a retrospective cohort study of 29,733 CRC patients, approximately 9% of deaths were attributable to congestive heart failure (CHF), more than 5% were attributable to chronic obstructive pulmonary disease (COPD), and nearly 4% were attributable to diabetes mellitus (DM) [10]. Several studies have also suggested that CRC patients burdened by comorbidities have higher mortality than those without coexisting diseases [8,11,12]. Thus, increasing the understanding about and attention to patterns of diseases that coexist with CRC is important for disease management and more personalized medicine.

To date, investigations on comorbidities associated with CRC have primarily focused on commonly used comorbidity indices, such as the Charlson comorbidity index and Elixhauser comorbidity index [5,13-15], or defined a comorbid condition as 1 of the 14 selected health conditions [16]. Although some comorbid conditions, such as hypertension and DM, are well recognized [5,15,17,18], many others are likely unidentified. A significant gap in knowledge remains regarding comorbidity patterns with CRC, especially among Chinese populations whose lifestyles and diets differ from those of Western populations.

In recent years, the development of network theory has provided new approaches to understand the complex interrelations between diseases [19-21]. The phenotypic comorbidity network (PCN) also known as the disease co-occurrence network has been widely used to study the comorbidity patterns of various chronic conditions, such as depression [22], ischemic heart disease (IHD) [23], pediatric pulmonary hypertension [24], migraine [25], COPD [26], hip fracture [27], and hepatocellular carcinoma [28]. Additionally, rapid growth in administrative health data (eg, hospital discharge data) offers opportunities to simultaneously assess the entire spectrum of diagnoses in comorbidity studies [29,30]. Administrative health data include a substantial amount of health information in the form of ICD-10 (International Classification of Diseases, 10th Revision) codes, which have tremendous potential to increase the understanding of the nature of comorbidities [30]. Nevertheless, to our knowledge, no study has yet applied network theory with regional administrative health data to systematically exploit the hidden information of comorbidity patterns in Chinese patients with CRC.

The aim of this study was to apply a network-based approach using routinely collected hospital discharge records (HDRs) to identify comorbidity patterns in a general population of newly diagnosed CRC patients. Comorbidity patterns were assessed by measuring the prevalence of individual diseases and the comorbid strength of coexisting diseases and by determining differences in sex, age at diagnosis, region, and cancer site.

## Methods

### Study Design and Participants

A retrospective cohort study was performed using longitudinal data from a provincial health administrative data set. The data set was mandatorily collected, and it included routinely collected anonymized HDRs from all secondary hospitals and tertiary hospitals in Sichuan Province, China, since 2015 [21]. HDRs are medical record documents with legal effect and cover detailed discharge diagnosis information, which are coded using standardized ICD-10 codes [31]. Each inpatient’s longitudinal clinical data were available, including anonymized identity, age, sex, residential address, admission date, discharge date, principal discharge diagnosis, and up to 15 secondary diagnoses. Sichuan Province, one of the most populous provinces in China, is separated into 2 cultural/topographic sections by the Hengduan Mountains, thus providing a scaled-down model of the broader situation in China [32,33]. 

**Figure 1:**
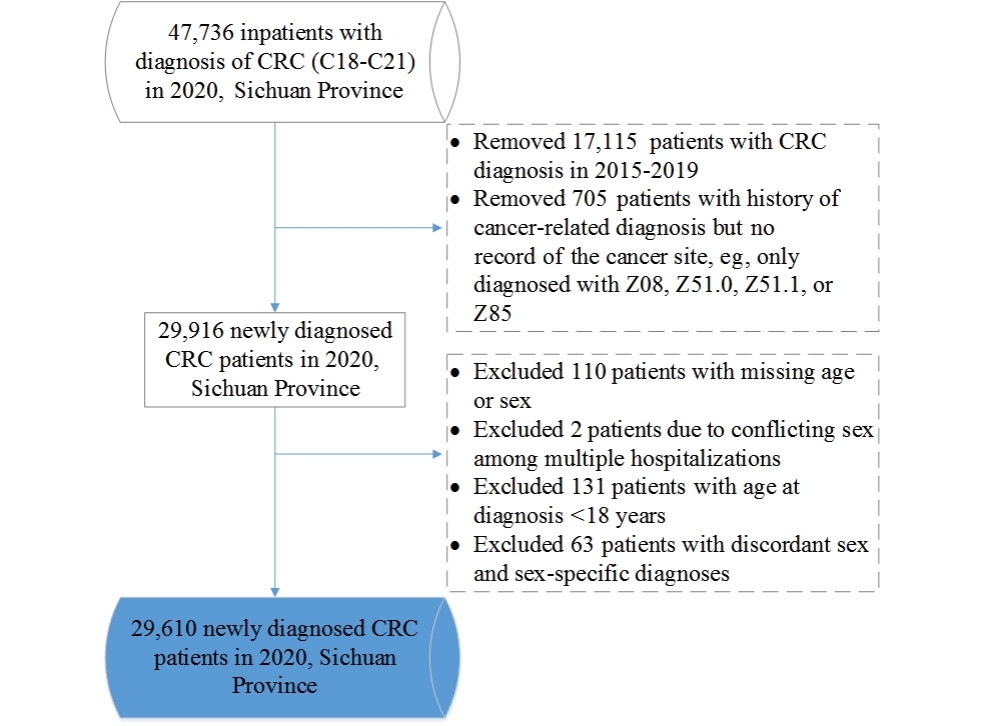
Flowchart of the study participants. CRC: colorectal cancer; Z08: follow-up examination after treatment for malignant neoplasms; Z51.0: radiotherapy session; Z51.1: chemotherapy session for neoplasm; Z85: personal history of malignant neoplasm.

A previous validation study integrated HDRs in Sichuan Province to identify new cancer cases, and indicated no statistical significance with findings from population-based cancer registry data, but it had important strengths, including a large sample, high accuracy of diagnosis coding, and time effectiveness [34]. In this study, provincial HDRs were used to identify new cases of CRC, where a new case was defined as a patient without any CRC diagnosis code (ICD-10 codes: C18 to C21) for at least 5 years before the first CRC diagnosis [17]. To minimize bias, all inpatients living in Sichuan Province during the study period were considered, with the exception of those with incomplete information or incorrect codes. The inclusion criteria were permanent residence in Sichuan Province, age ≥18 years at diagnosis, and first CRC occurrence between January 1, 2020, and December 31, 2020. Patients with incomplete information, conflicting reports of sex among multiple hospitalizations, conflicts between diagnoses and sex, or a history of a malignant neoplasm but with an unknown or not reported cancer site were excluded. Based on the longitudinal health care data, 29,610 patients diagnosed with first CRC occurrence were identified between January 1, 2020, and December 31, 2020 (Figure 1).

### Ethical Considerations

This study was approved by the Ethics Committee of the Health Information Center of Sichuan Province (ZX-EC202100301). The requirement of obtaining informed consent was waived because of the secondary nature of the deidentified data and the retrospective study design.

### Definition of Comorbidity

Because chronic conditions are not expected to end in a single hospitalization period, a 5-year look-back period from the first diagnosis was used to identify comorbidities [17]. First, for each CRC patient, an index hospitalization was identified, which was the first hospitalization that occurred or was within 4 weeks after the date of the first diagnosis of CRC [17]. Second, the index hospitalization and hospitalizations during the 5 years before the first diagnosis of CRC were scanned to determine the history of diseases. Lastly, the Chronic Condition Indicator, defined by the Healthcare Cost and Utilization Project, was used to differentiate between acute and chronic ICD-10 codes at 3 digits [20,35]. Diagnostic codes from chapters XV to XXII, which are certain conditions originating in pregnancy, childbirth, puerperium, and the perinatal period or are general symptoms, were excluded, as the study population included mainly middle-aged or older patients. Notably, to exclude the complications of CRC or its treatment, some chronic conditions that occurred in the index hospitalization were excluded [17], including secondary malignant neoplasm, anemia, nutritional marasmus, agranulocytosis, metabolism disorders, and intestinal obstruction. To estimate the effects of different look-back periods on comorbidity patterns, we performed a complementary analysis using a 3-year look-back period.

### Comorbidity Prevalence

The prevalence of all chronic diagnoses with ICD-10 codes and the corresponding 95% CIs were calculated. The prevalence cutoff was set to a statistically significant value of >1% (1-sided test, *P*<.025) in at least one subgroup to avoid diagnoses used very rarely or never [36]. The final list of 66 distinct comorbidities and their ICD-10 codes are provided in Multimedia Appendix 1. To assess differences in comorbidity prevalence by sex, region, and cancer site, relative differences in prevalence were calculated and then tested for significance using a Z-test with Bonferroni correction (α level of .05). With relative difference by sex as an example, the calculation was according to the following equation [20]:



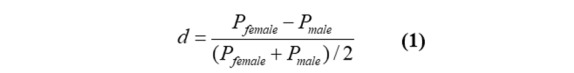



where *P_female_* and *P_male_* are the prevalences of comorbidities in females and males, respectively.

If the relative difference was greater than 0.1 [28] and the absolute difference was significant after Bonferroni correction, there was a significant difference in comorbidity prevalence. Among comorbidities with a significant difference in prevalence, enriched comorbidities were assessed further. When a comorbidity had a ≥0.5-fold increase in prevalence, it was defined as enriched. For example, a comorbidity was enriched in females when there was a significant prevalence difference by sex and a ≥0.5-fold increase in prevalence in females compared with males. A Cochran-Armitage trend test was applied to assess whether the prevalence of comorbidities increased with age. Furthermore, a K-means clustering algorithm was applied to cluster comorbidities in terms of prevalence by age. As the prevalence of comorbidities increased with age, we further adjusted the influence of age in estimating the enrichment comorbidities. The total number of cases in each age group (18-49, 50-59, 60-69, 70-79, and ≥80 years) by sex, region, and cancer site was regarded as the standard population.

### PCN Construction

A PCN was constructed to capture the coexistence of all comorbidities (also named connections) as recorded through HDRs. In a PCN, nodes represent chronic disease codes (ICD-10 codes at 3 digits) that are connected through edges. Node size is proportional to disease prevalence, and node color identifies the ICD-10 category. The cosine index was applied to quantify the comorbid strength of coexisting diseases, which considers the co-occurrence and prevalence of comorbidities and thus is not affected by the sample size [37]. To detect comorbidity coexistence measured by the cosine index, a cutoff was defined by assessing the relationship between the Pearson correlation and cosine index, in which the number of significant coexisting comorbidities was equal in both networks measured by the cosine index and Pearson correlation [21,38]. The cosine index and Pearson correlation coefficient were defined as in equations (2) and (3), respectively, and the significance of *ϕ_ab_*≠0 was determined by performing a *t* test, which was calculated according to equation (4).



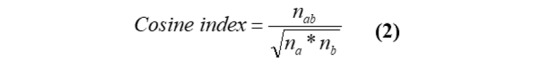





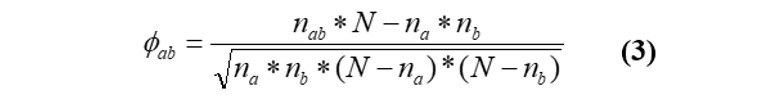





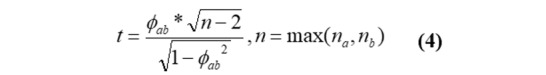



In the equations, *N* is the total number of newly diagnosed CRC patients, and *n_a_*, *n_b_*, and *n_ab_* denote the number of patients with disease *a*, disease *b*, and both diseases, respectively.

### Network Properties

The structural properties of a PCN are measured using network indices, such as network density, degree, average degree of neighbors, and betweenness centrality [39]. Network density is the proportion of significant connections to all possible connections in a network, which measures how compact a network is. A higher network density is associated with a higher number of connections between comorbidities. The number of correlations with other comorbidities is degree (*k*), which provides a general idea of how involved a comorbidity is with other comorbidities. Median and IQR were calculated to describe the distribution of degree (*k*). A Kolmogorov-Smirnov test was applied to estimate whether the degree (*k*) distribution followed a power law. When one comorbidity was directly connected with other comorbidities, the others were named as neighbors. The average degree of neighbors was calculated to measure neighbor connectivity. Betweenness centrality, which is the number of shortest paths between any 2 nodes of which a node is a part divided by all possible paths, indicates how central one comorbidity is relative to all other comorbidities. A high betweenness centrality indicates a high likelihood of forming bridges between other comorbidities or the end points of many comorbidities [25]. To identify the most important comorbidity in a PCN, the PageRank algorithm that considers the edge weight was applied [36]. A higher PageRank value of a comorbidity is associated with a greater effect on the network. Comorbidities with the top 10 percentiles of PageRank values were defined as the most important comorbidities in PCNs.

### Abundant Comorbidity Connections in Each Subgroup of CRC Patients

PCNs were constructed separately for males and females, for rural and urban patients, and for rectal and colon cancer patients. Then, comorbid strengths were compared to measure disparities by sex, region, and cancer site. When a coexisting disease was unique to or enriched (with 0.05 higher comorbid strength than another) in a given subgroup, the difference was notable and thus was defined as an abundant connection. With sex as an example, when a comorbidity connection occurred only in males or occurred in both subgroups but the comorbid strength was 0.05 (approximately half of the minimum comorbid strength in the PCN) higher in males than in females, the comorbidity connection was abundant in males.

All analyses and visualizations were conducted using R 4.0.3 (R Foundation for Statistical Computing).

## Results

### Characteristics of Newly Diagnosed CRC Patients

**Figure 2:**
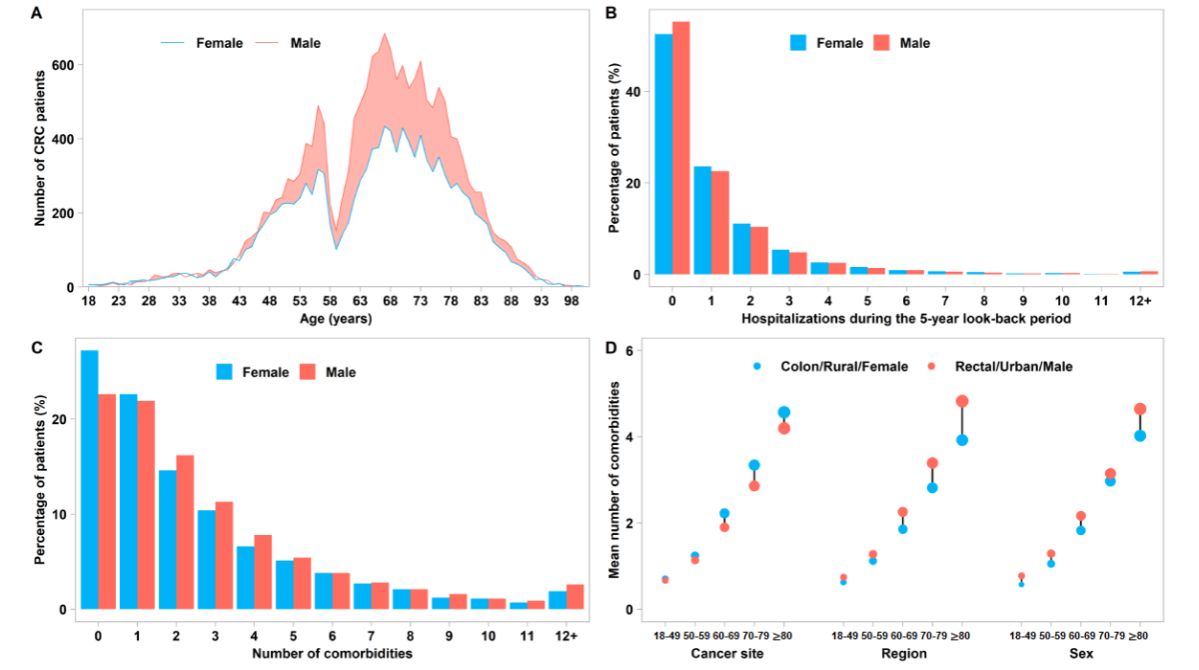
Number of comorbidities and hospitalizations for newly diagnosed colorectal cancer (CRC) patients in 2020 in Sichuan Province, China. (A) Age at diagnosis by sex. (B) Hospitalizations during the 5-year look-back period before diagnosis. (C) Frequency of patients per distinct number of comorbidities per patient among male and female patients. (D) Age-specific mean number of comorbidities by cancer site, region, and sex.

Table 1 shows the characteristics of the 29,610 newly diagnosed CRC patients. The mean patient age at diagnosis was 65.6 years. Among the patients, 30.1% (8922/29,610) were diagnosed at <60 years of age, 58.9% (17,426/29,610) were male, 45.7% (13,522/29,610) lived in urban regions, and 52.5% (15,543/29,610) had rectal cancer. Overall, approximately one-fourth (7241/29,610, 24.5%) of patients were without any comorbidity, 22.2% (6574/29,610) had 1 comorbidity, and more than half (15,795/29,610, 53.3%) had ≥2 comorbidities. The sex-specified distributions of age at diagnosis, number of hospitalizations during the 5-year look-back period, and number of comorbidities are presented in Figure 2A-C. The frequency of at least one comorbidity was significantly higher in male patients than in female patients (13,495/17,426, 77.4% vs 8874/12,184, 72.8%; *P*<.001), in urban patients than in rural patients (10,542/13,522, 78.0% vs 11,746/15,988, 73.5%; *P*<.001), and in colon cancer patients than in rectal cancer patients (10,115/13,064, 77.4% vs 11,528/15,543, 74.2%; *P*<.001). The proportion of patients with at least one comorbidity increased with age (Cochran-Armitage trend test, *P*<.001), and the increasing trend was similar in each subgroup. The mean number of comorbidities by cancer site, region, and sex increased with age (Figure 2D).

**Table 1:** Descriptive characteristics of patients newly diagnosed with colorectal cancer in 2020 in Sichuan Province, China.

Demographic and clinical factors		Value (N=29,610)
Age at diagnosis (years), mean (SD)		65.6 (12.9)
**Age group at diagnosis (years), n (%)**		
	18-49	3384 (11.4)
	50-59	5538 (18.7)
	60-69	8308 (28.1)
	70-79	8582 (29.0)
	≥80	3798 (12.8)
**Sex, n (%)**		
	Female	12,184 (41.1)
	Male	17,426 (58.9)
**Region, n (%)**		
	Rural	15,988 (54.0)
	Urban	13,522 (45.7)
	Unknown	100 (0.3)
**Cancer site, n (%)**		
	Colon	13,064 (44.1)
	Rectum	15,543 (52.5)
	Anus	813 (2.7)
	Multiple sites	190 (0.6)
**Number of comorbidities, n (%)**		
	0	7241 (24.5)
	1	6574 (22.2)
	2	4595 (15.5)
	3	3241 (10.9)
	4	2172 (7.3)
	5	1563 (5.3)
	≥6	4224 (14.3)

### Comorbidity Prevalence and Differences Between Subgroups

Among the 66 distinct comorbidities (Multimedia Appendix 1), the most prevalent comorbidities were hypertension (8581/29,610, 29.0%; 95% CI 28.5%-29.5%), hyperplasia of the prostate (3816/17,426, 21.9%; 95% CI 21.3%-22.5%), COPD (4199/29,610, 14.2%; 95% CI 13.8%-14.6%), DM (3715/29,610, 12.5%; 95% CI 12.2%-12.9%), and IHD (3200/29,610, 10.8%; 95% CI 10.5%-11.2%). Comorbidity prevalence using a 3-year look-back period is shown in Multimedia Appendix 2, and a scatter plot of comorbidity prevalence using 3-year and 5-year look-back periods is shown in Multimedia Appendix 3. As for subgroup difference, most comorbidities were common in each subgroup, whereas a few comorbidities were only detected in a subgroup and with relatively low prevalence. For example, anxiety disorder (ICD-10: F41) was only detected in females, with a prevalence of 1.3% (156/12,184; 95% CI 1.1%-1.5%). Among comorbidities with significant differences in prevalence (Multimedia Appendices 4-6), differences were large and some comorbidities were enriched (prevalence increased by ≥50%) in a subgroup (Table 2). For example, COPD (ICD-10: J43 and J44), renal failure (N18 and N19), conduction disorders (I44 and I45), and gout (M10) were enriched in males, whereas osteoporosis (M81), gonarthrosis (M17), other nontoxic goiter (E04), and anxiety disorder (F41) were enriched in females. Most comorbidities had higher prevalence and were even enriched (prevalence increased by ≥50%) in urban patients, for example, atherosclerosis (ICD-10: I70), cerebrovascular diseases (I65 and I67), hypertensive heart disease (I11), diverticular disease of the intestine (K57), and spondylosis (M47). Comorbidities had somewhat higher prevalence in colon cancer patients than in rectal cancer patients, and disorders of glycoprotein metabolism (ICD-10: E77), other coagulation defects (D68), angina pectoris (I20), other diseases of the biliary tract (K83), and malignant neoplasm of the liver and intrahepatic bile ducts (C22) were enriched in colon cancer patients. After adjustment with age, osteoporosis (ICD-10: M81) was not enriched in urban patients, and the other comorbidities still showed enrichment (Multimedia Appendix 7).

**Figure 3:**
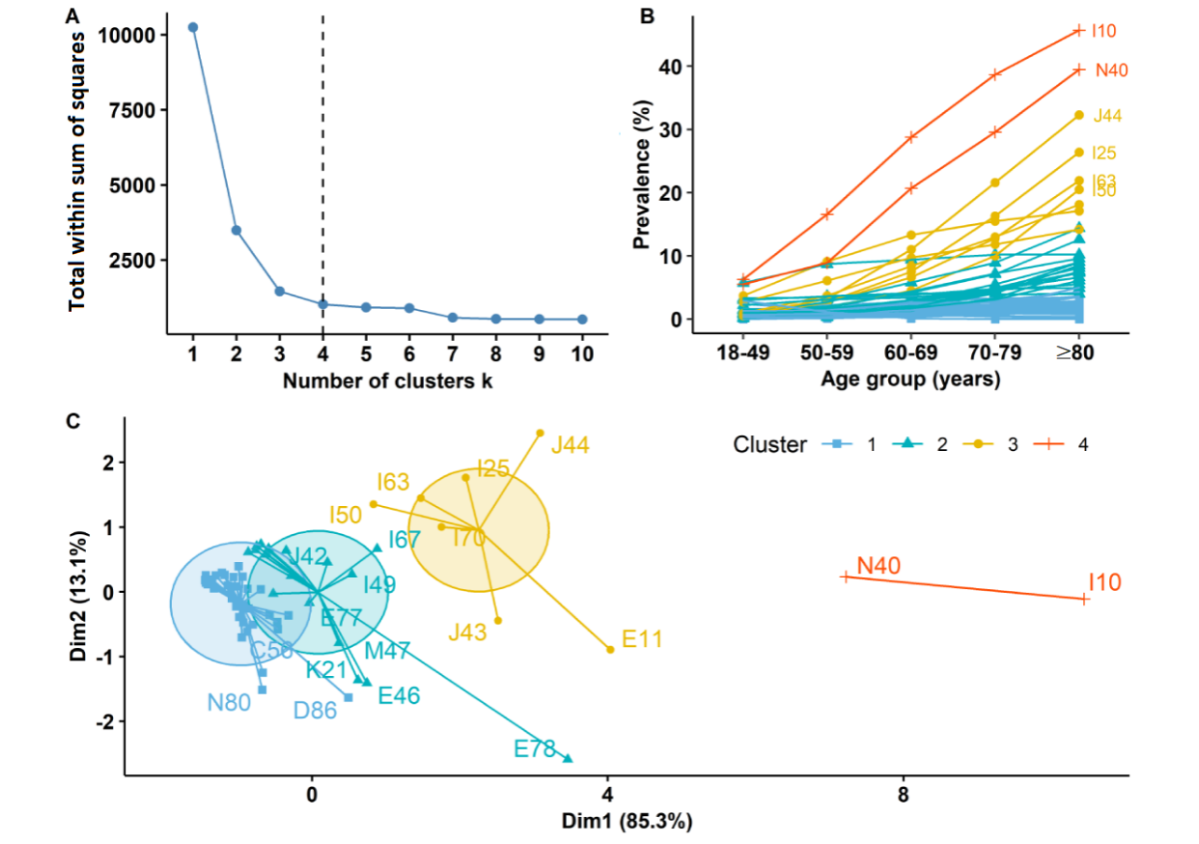
Clusters of comorbidities in colorectal cancer patients based on age-specific prevalence in Sichuan Province, China. (A) Optimal number of clusters using K-means clustering algorithm. (B) Age-specific prevalence of comorbidities in each cluster. Here, comorbidities with a prevalence of >20% in the ≥80 years age group were labeled with ICD-10 (International Classification of Diseases, 10th Revision) codes at 3 digits. (C) Cluster plot. Based on the principal component analysis, comorbidity prevalence in 5 dimensions (18-49, 50-59, 60-69, 70-79, and ≥80 years) was reduced to 2 dimensions (x-lab and y-lab). The ICD-10 codes are clarified in Multimedia Appendix 1.

For age-specific comorbidities, 62 comorbidities with a prevalence significantly greater than 1% in at least one age group were divided into 4 clusters (Figure 3). Comorbidity prevalence increased with age, but the rate of increase varied by cluster. Cluster 1 contained 36 comorbidities with low prevalence (the average prevalence among the 5 age groups ranged from 0.8% to 2.2%), and most had a low growth rate with age, but a few decreased with age (eg, endometriosis) or were stable across ages (eg, chronic sinusitis). Cluster 2 contained 17 comorbidities with moderate prevalence (the average prevalence among the 5 age groups ranged from 1.3% to 8.1%), and most had a relatively high growth rate with age. Cluster 3 contained 7 highly prevalent comorbidities, most of which had a high growth rate with age (the average prevalence among the 5 age groups ranged from 1.4% to 21.5%), including COPD (ICD-10: J44 and J43), IHD (I25), cerebral infarction (I63), heart failure (I50), atherosclerosis (I70), and DM (E11). Cluster 4 contained the 2 comorbidities with the highest prevalence (hypertension [ICD-10: I10] and hyperplasia of the prostate [N40]).

**Table 2:** Absolute prevalence differences of enrichment comorbidities in subgroups of colorectal cancer patients in Sichuan Province, China.

ICD-10 code	Enrichment comorbidity	Prevalence, value (95% CI)	Absolute prevalence difference, value (95% CI)^a^		
			Male-female	Urban-rural	Colon-rectal
J44	COPD^b^	14.2 (13.8 to 14.6)	7.9 (7.2 to 8.7)	—^c^	—
J43	Emphysema	9.4 (9.1 to 9.8)	6.9 (6.3 to 7.5)	—	—
I70	Atherosclerosis	9.2 (8.9 to 9.6)	—	4.2 (3.5 to 4.9)	—
I67	Other cerebrovascular diseases	6.7 (6.4 to 6.9)	—	3.6 (3.0 to 4.2)	—
K21	Gastroesophageal reflux disease	3.8 (3.6 to 4.0)	—	2.3 (1.9 to 2.8)	—
M47	Spondylosis	3.8 (3.6 to 4.0)	—	1.7 (1.2 to 2.1)	—
E77	Disorders of glycoprotein metabolism	3.6 (3.4 to 3.8)	—	—	1.8 (1.3 to 2.2)
M81	Osteoporosis without pathological fracture	3.1 (2.9 to 3.3)	−3.5 (−3.9 to −3.0)	—	—
I11	Hypertensive heart disease	3.0 (2.8 to 3.2)	—	1.6 (1.2 to 2.0)	—
N19	Unspecified renal failure	2.5 (2.3 to 2.7)	1.2 (0.9 to 1.6)	—	—
E04	Other nontoxic goiter	2.2 (2.0 to 2.3)	−2.0 (−2.3 to −1.6)	1.1 (0.7 to 1.4)	—
D68	Other coagulation defects	2.1 (1.9 to 2.2)	—	—	1.0 (0.7 to 1.4)
K83	Other diseases of the biliary tract	2.1 (2.0 to 2.3)	—	—	0.9 (0.6 to 1.3)
I65	Occlusion and stenosis of precerebral arteries	1.9 (1.8 to 2.1)	—	1.1 (0.8 to 1.4)	—
N18	Chronic renal failure	1.7 (1.6 to 1.9)	0.7 (0.4 to 1.0)	1.1 (0.8 to 1.4)	—
K57	Diverticular disease of the intestine	1.5 (1.4 to 1.6)	—	1.5 (1.4 to 1.6)	—
M17	Gonarthrosis	1.5 (1.4 to 1.7)	−1.3 (−1.6 to −1.0)	0.8 (0.5 to 1.1)	
I20	Angina pectoris	1.3 (1.2 to 1.4)	—	0.7 (0.5 to 1.0)	0.6 (0.4 to 0.9)
I44	Atrioventricular and left bundle-branch block	1.3 (1.2 to 1.4)	0.8 (0.5 to 1.0)	—	—
I45	Other conduction disorders	1.2 (1.1 to 1.4)	0.7 (0.5 to 1.0)	—	—
M10	Gout	1.2 (1.1 to 1.3)	1.5 (1.3 to 1.7)	0.6 (0.3 to 0.8)	—
E27	Other disorders of the adrenal gland	1.1 (1.0 to 1.3)	—	0.7 (0.5 to 0.9)	—
C22	Malignant neoplasm of the liver and intrahepatic bile ducts	1.1 (1.0 to 1.3)	—	—	0.5 (0.2 to 0.7)
F41	Other anxiety disorders	0.8 (0.7 to 0.9)	−0.8 (−1.1 to −0.6)	—	—

^a^Absolute prevalence differences by sex, region, and cancer site were statistically significant after Bonferroni correction.

^b^COPD: chronic obstructive pulmonary disease.

^c^The comorbidity was not enriched in this subgroup.

### PCN in Newly Diagnosed CRC Patients

**Figure 4:**
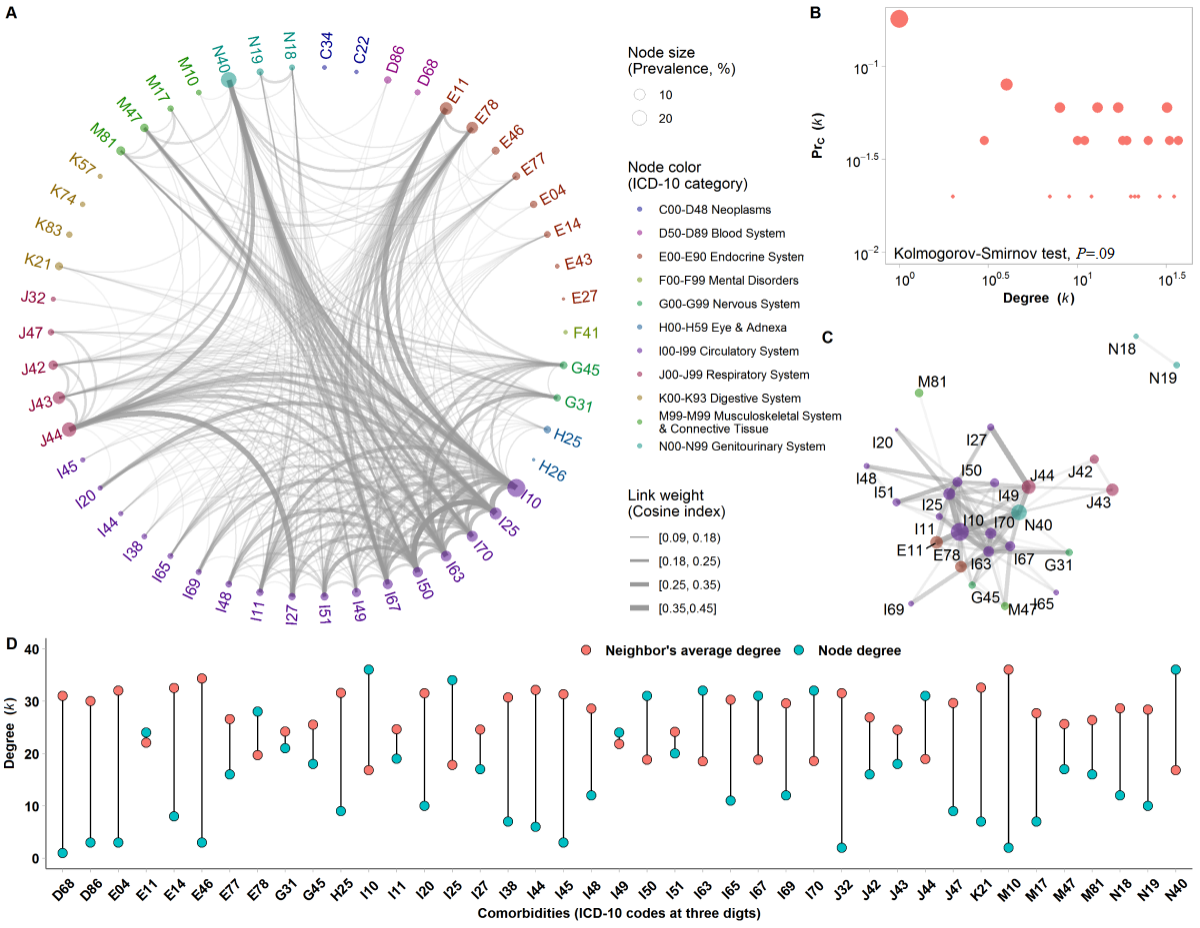
Phenotypic comorbidity network (PCN) in newly diagnosed colorectal cancer (CRC) patients in Sichuan Province, China. (A) The PCN in newly diagnosed CRC patients. Nodes represent comorbidities (ICD-10 [International Classification of Diseases, 10th Revision] codes at 3 digits), such that the node size is proportional to the comorbidity prevalence in CRC patients and its color identifies the ICD-10 category. Link weights are proportional to the magnitudes of the cosine index. (B) Cumulative degree (k) distribution. The degree distribution showed an exponential decay when the degree was ≥12 (Kolmogorov-Smirnov test, *P*=.09). (C) The top 100 edges in the PCN. Here, the top 100 links where cosine index values were ≥0.18 are shown. (D) The degree distribution of the node and its neighbors. Here, the connected nodes (degree >0) in the PCN are shown. The ICD-10 codes are clarified in Multimedia Appendix 1.

The PCN of newly diagnosed CRC patients contained 50 diseases and 327 significant comorbid disease pairs (Figure 4). The number of significant correlations shared with other comorbidities per diagnosis code was 10.5 (IQR 3.0-19.8). Hypertension (ICD-10: I10), IHD (I25), hyperplasia of the prostate (N40), cerebral infarction (I63), and heart failure (I50) were the most important comorbidities in the PCN, indicating that they often coexisted with one another, had the highest number of coexisting comorbidities, and thus largely increased the complexity of coexisting diseases. Among the top 100 significant comorbid disease pairs with a cosine index ≥0.18, 51 connections occurred within the same disease system (eg, 45 connections occurred within circulatory system diseases) and 49 connections involved 2 disease systems. Across betweenness centrality measures, comorbidities showed the highest values in disorders of lipoprotein metabolism (ICD-10: E78), hyperplasia of the prostate (N40), COPD (J44), other cerebrovascular diseases (I67), and heart failure (I50). The results indicated that those comorbidities mediated correlations between others or were the end points of many comorbidities. When those 5 comorbidities (10% of comorbidities in the PCN) were removed, the scale of the PCN decreased considerably, with the number of significant comorbid disease pairs decreasing by 45%. Notably, hypertensive heart disease (ICD-10: I11), one of the 30 comorbidities with fewer coexisting diseases than their neighbors, connected with all 10 of the most important diseases and thus ranked as the 11th most important disease in the network.

### Differences in PCNs Between Subgroups

Table 3 shows the network structures of PCNs constructed separately for males and females, for different age groups, for rural and urban patients, and for rectal cancer patients and colon cancer patients. The number of comorbidities with a prevalence of >1% in early onset CRC patients (diagnosis age of 18-49 years) was 15, and comorbidities were not significantly connected. Comorbidity coexistence relationships were more complex in males than females, older patients than those aged 50-59 years, and urban patients than rural patients. Male and female patients had 46 and 43 comorbidities, respectively, with median numbers of connections for comorbidities of 10.5 (IQR 2.0-17.5) and 6 (IQR 0.5-12.5), respectively, and median numbers of connections for node’s neighbors of 23.2 (IQR 19.0-25.5) and 17.9 (IQR 15.0-19.0), respectively. Except in the subgroup of CRC patients with a diagnosis age of 50-59 years, the most important comorbidities in the PCNs were similar, with hypertension (ICD-10: I10), IHD (I25), and cerebral infarction (I63) common to each subgroup. In early onset CRC patients (diagnosis age of 18-59 years), the most important comorbidities in the PCN were hypertension (ICD-10: I10) and disorders of lipoprotein metabolism (E78).

**Figure 5:**
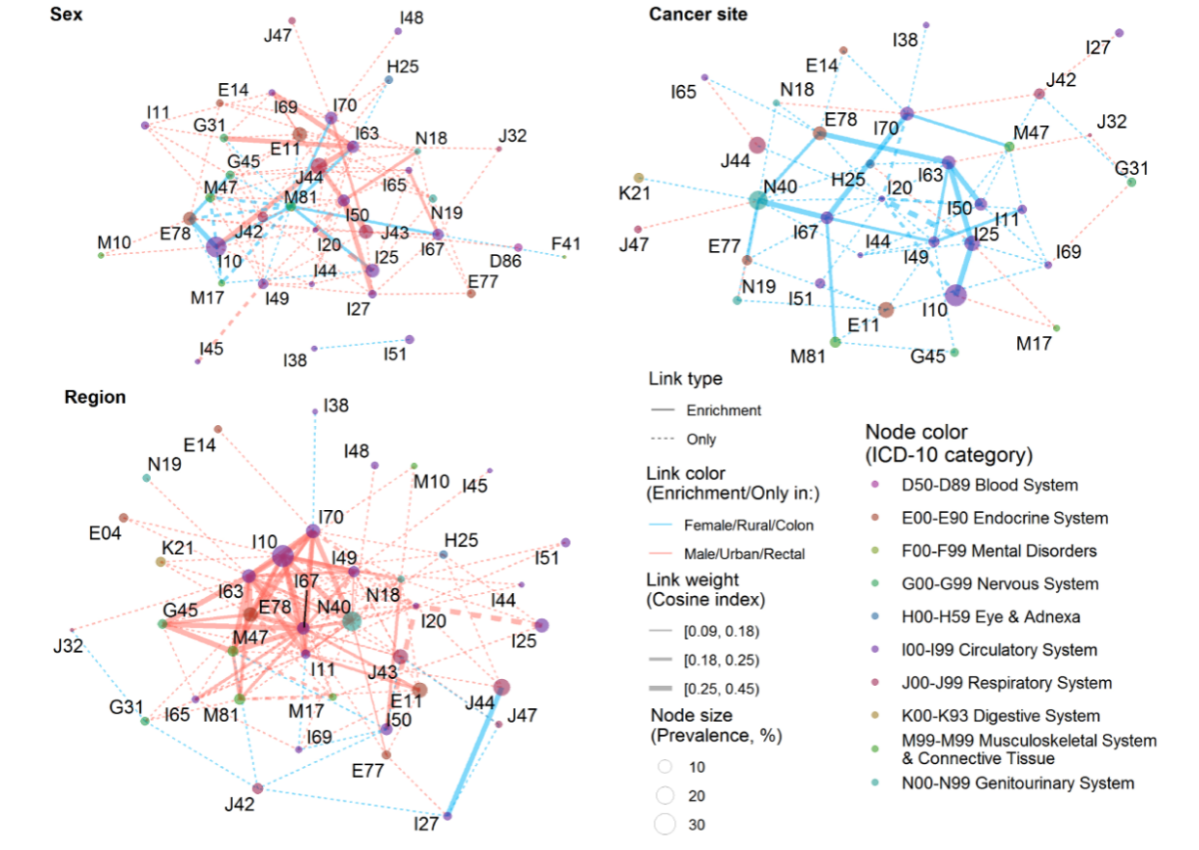
Abundant connections by sex, region, and cancer site in newly diagnosed colorectal cancer patients in Sichuan Province, China. Except for those common to both subgroups (link weight difference <0.05), disease pairs were identified as abundant edges in one subgroup, including enrichment (solid lines) or only occurrence (dotted lines). For example, if the link weight difference by sex (female-male), region (rural-urban), and cancer site (rectal-colon) was ≥0.05, the disease pair was enriched in females, rural patients, and colon cancer patients, and the link was colored blue. On the other hand, if the difference was ≤−0.05, the disease pair was enriched in males, urban patients, and rectal cancer patients, and the link was colored red. The ICD-10 (International Classification of Diseases, 10th Revision) codes are clarified in Multimedia Appendix 1.

Figure 5 shows the abundant connections in a given subgroup of newly diagnosed CRC patients. Without considering sex-specific diseases, 141 connections were common to both sexes, but 9 and 7 connections were separately enriched in males and females (cosine index increased more than 0.05), respectively, and 71 and 14 connections were significant only in males and females, respectively. Abundant connections in females were primarily associated with osteoporosis (ICD-10: M81) or gonarthrosis (M17), whereas abundant connections in males were primarily associated with COPD (J42, J43, J44, and J47), cerebrovascular diseases (I65 and I69), atherosclerosis (I70), heart failure (I50), or renal failure (N18 and N19). Urban-rural disparities in comorbidity connections were very large, with 13 (13/260, 5%) and 117 (117/260, 45%) abundant connections in rural and urban patients, respectively. Abundant connections in urban patients were primarily associated with disorders of lipoprotein metabolism (ICD-10: E78), DM (E11), hypertension (I10), hypertensive heart disease (I11), IHD (I20 and I25), cardiac arrhythmias (I44, I48, and I49), cerebrovascular diseases (I63, I65, I67, and I69), atherosclerosis (I70), gonarthrosis (M17), spondylosis (M47), osteoporosis (M81), renal failure (N18), or hyperplasia of the prostate (N40). Differences in connections according to cancer site were relatively small, with 181 (181/250, 72.4%) connections common to both colon cancer and rectal cancer patients.

**Table 3:** Phenotypic comorbidity network structures in subgroups of newly diagnosed colorectal cancer patients in Sichuan Province, China.

Subgroup		Nodes, n	Density	Degree, median (IQR)	Degree of neighbors, median (IQR)	Most important diseases in the PCN^a,b^
**Sex**						
	Female	43	0.189	6.0 (0.5-12.5)	17.9 (15.0-19.0)	I10, I25, I63, and I50
	Male	46	0.251	10.5 (2.0-17.5)	23.2 (19.0-25.5)^c^	I10, I63, N40, and I25
**Age group (years)**						
	18-49	15	0	0	0	—^d^
	50-59	26	0.043	0 (0-1.1)	3.0 (2.8-3.4)	E11 and I10
	60-69	42	0.099	1.5 (0-7.8)^c^	11.2 (8.5-13.5)^c^	I10, I63, and I50
	70-79	52	0.122	3.0 (0-11.0)^c^	17.1 (13.3-19.2)^c^	I10, I63, I25, and N40
	≥80	58	0.099	1.0 (0-9.8)^c^	18.6 (13.5-21.0)^c^	I10, N40, I63, and I25
**Region**						
	Rural	46	0.178	6.0 (0-12.8)	18.5 (15.1-20.0)	I10, I25, I50, and I63
	Urban	50	0.202	8.0 (1.0-15.8)	21.7 (18.4-24.7)^c^	I10, I63, N40, and I25
**Cancer site**						
	Rectal	44	0.218	8.5 (1.0-14.0)	19.1 (16.6-20.3)	I10, I25, I63, and I50
	Colon	52	0.179	7.0 (0-16.0)	20.3 (17.6-23.0)	I10, I25, N40, and I63

^a^PCN: phenotypic comorbidity network.

^b^Comorbidities with the top 10 percentiles of PageRank values were defined as the most important comorbidities in the PCN, and they were sorted by descending PageRank values. The ICD-10 codes and corresponding comorbidities were are follows: I10, hypertension; I25, chronic ischemic heart disease; I63, cerebral infarction; I50, heart failure; N40, hyperplasia of the prostate; E78, disorders of lipoprotein metabolism.

^c^Greater average degree of nodes or average degree of neighbors in males, older patients, and urban patients than in females, patients aged 50-59 years, and rural patients, respectively (1-sided *t* test with *P*<.025).

^d^Comorbidities were not connected to colorectal cancer patients aged 18-49 years at diagnosis.

## Discussion

### Principal Findings

In this population-based cohort study, the entire spectrum of comorbidities in patients newly diagnosed with CRC was examined by scanning all hospitalizations during a 5-year look-back period before the first CRC diagnosis. Comorbidities were common in CRC patients at diagnosis, and prevalence and complex coexistence relationships varied by sex, age at diagnosis, region, and cancer site. The most prevalent comorbidities were hypertension, hyperplasia of the prostate, COPD, DM, and IHD. There were large disparities in disease co-occurrences in the male-female and urban-rural subgroups. Prevalence, diversity of diseases, and strength of disease coexistence were higher in urban patients, whereas severe diseases were more likely to co-occur in rural patients, such as the sequelae of cerebrovascular disease or COPD co-occurring with heart failure.

It was not surprising that comorbidities were common among newly diagnosed CRC patients, because both CRC and chronic diseases generally occur in older people [40-42]. CRC and comorbid conditions, such as DM and hypertension, may share common risk factors, such as smoking, poor diet, hyperlipidemia, lack of physical activity, and obesity [43-45]. In this population-based study, the most prevalent comorbidities were hypertension, hyperplasia of the prostate, COPD, DM, and IHD. Similar results were found in previous studies in New Zealand and the United States, although the prevalence of comorbidities differed [8,17,46]. Variations of prevalence might be related to differences in the study population (eg, some studies focused only on elderly patients among whom comorbidities are more common) and the definition used for chronic conditions (eg, a 1-year look-back period for hospitalization data resulted in a lower yield of comorbid conditions). Accumulating evidence suggests that sedentary lifestyle, metabolic syndrome (presence of ≥3 conditions among obesity, hypertension, hyperlipidemia, and DM), and antibiotic consumption may contribute to the development of early onset CRC [45,47-49]. In our study, we found 15 comorbidities with prevalences greater than 1% in early onset CRC patients, where hypertension, disorders of lipoprotein metabolism, hyperplasia of the prostate, and DM were the most prevalent comorbidities. Comorbidity is a strong and independent factor in the treatment and survival of CRC patients [5,50,51], for example, CRC patients comorbid with DM continued to receive chemotherapy less frequently than those without DM. Given the survival disadvantage in CRC patients with comorbidities [52], further evidence on the risks and benefits of therapy according to the types of co-occurring comorbidities and the severity of the diseases is needed to foster personalized medical care for those patients.

The complex coexistence relationships that occur among comorbidities are not due to simple coincidence, and therefore, to understand those relations, comorbidities must be studied collectively and not in isolation. Coexisting diseases within the same disease system were primarily within the circulatory system. The highest number of comorbidities was within the circulatory system, and those comorbidities included the highly prevalent diseases of hypertension, IHD, heart failure, and atherosclerosis. Coexisting diseases involved 2 disease systems, and they primarily included diseases of the circulatory system and those of the endocrine, nervous, respiratory, genitourinary, musculoskeletal, or connective tissue system. In a population-based cohort study in Spain, the most frequent comorbidity was DM, and the most frequent disease pair was CHF with DM, followed by CHF with COPD and peripheral vascular disease with DM [53]. In that study, the modified Charlson comorbidity score (only including 12 comorbidities) used to measure comorbidity might have underestimated the comorbidity burden and the complex coexistence relationships, because it did not include some important comorbidities, such as highly prevalent hypertension (I10). According to the network approach in this study, disorders of lipoprotein metabolism and hyperplasia of the prostate were part of some of the shortest paths between 2 comorbidities (mediated correlations between others), and heart failure was the end point of many comorbidities. Those comorbidities had a high likelihood to form bridges between other comorbidities, suggesting possibilities for the management of target comorbidities in CRC patients.

Many disease pairs were unique to or enriched in a sex group. In this regard, coexistence with COPD, cerebrovascular diseases, atherosclerosis, heart failure, or renal failure was predominant in males, whereas coexistence with osteoporosis or gonarthrosis was predominant in females. The prevalence of musculoskeletal disorders was higher in women than in men, resulting in an increased likelihood of co-existence with other highly prevalent chronic diseases (eg, hypertension, IHD, cerebrovascular diseases, or atherosclerosis) in female CRC patients. Notably, the prevalence of some severe diseases (eg, cerebral infarction and heart failure) was similar in males and females, but there were sex differences in coexistence with other chronic diseases (eg, a higher likelihood of cerebral infarction comorbid with the sequelae of cerebrovascular disease, COPD, or degenerative diseases of the nervous system, and heart failure comorbid with other pulmonary heart diseases or renal failure in males). Previous studies have found a prevalence difference in comorbidities by sex among CRC patients [54]. A population-based study using administrative data to describe comorbidities in CRC patients at diagnosis in Spain showed that the prevalence of COPD in males was 23.0%, which was 2.4 times higher than that in females (prevalence of 9.5%) [54]. In China, the prevalence of COPD in males was 19.0% (95% CI 16.9%-21.2%), which was approximately 2.3 times higher than that in females (prevalence of 8.1%, 95% CI 6.8%-9.3%), mainly because of a significant difference in smoking status between males and females (current smokers: 58.2% vs 4.0%) [55]. Such differences might indicate a disparity in lifestyle-related diseases, which may be due to physical, hormonal, or even genetic differences [56]. It is becoming increasingly standard practice to report sex-specific estimates because of the vital importance of gender consideration in precision medicine [56-58].

Notably, sex differences were observed in the prevalence of anxiety and the comorbid strength of anxiety with other diseases, for example, anxiety was enriched in females and had a higher likelihood of co-existence with cerebrovascular disease. Previous observational studies indicated that cardiocerebrovascular modulation and dynamic cerebral autoregulation are compromised in patients with anxiety [59-61]. In addition, evidence from a prospective cohort study indicated that greater anxiety or depression can not only impede adherence to healthy habits in CRC patients, but also be a marker for accelerated CRC progression [62]. Furthermore, in a previous population-based study in New Zealand, the prevalence of anxiety was 1.0% and 0.6% in colon cancer and rectal cancer patients, respectively [17], which were values similar to those in this study. The use of regional hospitalizations to identify the prevalence of clinically significant anxiety as well as other mental disorders might lead to underestimates, because estimated prevalence is generally higher when questionnaires are used [63,64]. Considering the well-known underestimated prevalence of mental disorders, especially in low- and middle-income countries [65], and their negative effects on quality of life, scanning for symptoms of anxiety and depression, implementation of dietary and physical activity interventions, and implementation of social support are of utmost importance among CRC patients at diagnosis and even years after treatment [66-68].

Rural-urban disparities were detected in the prevalence of comorbidities and the comorbid strength among comorbidities. Socioeconomic differences in the prevalence of comorbidities in CRC patients were also reported in England, where at least 11 of the 14 chronic conditions showed increasing prevalence along with the level of deprivation in CRC patients [16]. For example, the most deprived groups had approximately twice the odds of having COPD compared with the least deprived groups, which might be related to the higher prevalence of smoking in the more deprived population, but the association between smoking status and deprivation was not quantifiable as there was no information on smoking prevalence [16]. In our study, the prevalence of most comorbidities among newly diagnosed CRC patients was higher in urban patients than in rural patients, including some highly prevalent comorbidities, such as DM, disorders of lipoprotein metabolism, cerebrovascular diseases, atherosclerosis, and hyperplasia of the prostate, as well as some comorbidities with relatively low prevalence, such as nontoxic goiter, degenerative diseases of the nervous system, hypertensive heart disease, gout, and renal failure. Thus, comorbidities were more closely connected in urban patients than in rural patients, and some disease pairs were unique to or enriched in urban patients. Such differences might be associated with Western lifestyles in urban areas. Compared with the rural lifestyle, the urban lifestyle is more sedentary, with manual labor replaced by computer-based work and walking replaced by driving automobiles. Differences were also likely due to disparities in accessibility to diagnostic and treatment services between urban and rural residents [69,70]. Notably, the prevalences of hypertension, heart failure, and COPD were somewhat higher but without clinical significance in urban patients than in rural patients; whereas the sequelae of cerebrovascular disease comorbid with hypertensive heart disease or heart failure, heart failure comorbid with COPD or spondylosis, and COPD comorbid with pulmonary heart diseases were over-presented in rural patients. The co-occurrence of severe diseases in rural patients might be associated with relatively low adherence to colonoscopy screening among rural residents. Thus, CRC at diagnosis was generally more severe, with a significantly higher risk of diagnosis at a late stage [71]. Given the relatively low participation rate in colonoscopy screening in high-risk populations in China [72] and the coexistence of severe diseases at diagnosis, health promotion campaigns and adoption of noninvasive screening tests should be priorities to improve adherence and diagnosis in population-based programs.

We found that approximately 50% of comorbidities had significantly higher prevalence in colon cancer patients than in rectal cancer patients. A literature review indicated that diet, smoking, and physical activity might have different effects on colon cancer compared with the effects on rectal cancer, for example, physical activity decreased the risk of colon cancer but not of rectal cancer [73]. A population-based register of all primary cancers diagnosed in New Zealand was linked to routine hospital discharge data, and the comorbidity prevalence in colon and rectal cancer patients was identified, with a prevalence difference by cancer site similar to that in our study [17]. A previous study identified differences in patient characteristics and surgical outcomes between colon cancer and rectal cancer [74], and there was a significantly higher proportion of comorbidities in colon cancer patients than in rectal cancer patients (76.3% vs 68.8%; *P*=.02). Among CRC patients in the Central Region of Denmark in 2000-2011, 38% of colon cancer patients and 32% of rectal cancer patients had at least one Charlson comorbidity [75]. These previous studies reported the comorbidity difference by cancer site, but they did not provide a clear definition of comorbidity [74] or just focused on limited chronic diseases [75]. Further studies are needed to systematically explore the impacts of comorbidity patterns or comorbidity trajectories on the treatment choice and survival prediction in patients with colon and rectal cancers.

### Limitations

This study had some limitations. First, the results should be interpreted in the context of an inpatient population in a developing country, since the HDRs did not include outpatient records and mental conditions might not be consistently diagnosed and recorded in HDRs [22]. Moreover, Berkson bias is unavoidable when only using an inpatient population to estimate morbidity [76]. Second, stage information is not yet included in HDRs because of the inherent limitation of the administrative database [31,34]. Further studies are needed to explore differences in comorbidity patterns according to TNM stages. Third, we systematically analyzed all chronic conditions and comorbidity patterns in newly diagnosed CRC patients in different subgroups, but did not explore CRC-specific comorbidities. As the emergence of comorbidities increases with age, further studies are needed to explore comorbidities that can be attributed to CRC rather than age, which may be helpful to provide etiological hypotheses or to construct surveillance programs [77]. Lastly, the common comorbidity profile identified in the study appears to be associated with treatment decision-making; however, limited by the available data, the relationships between comorbidities with different disease severities and different outcomes could not be identified in our study. The provincial data set was implemented in 2015 and did not contain proactive patient follow-up. Population-based cancer registries in China mainly report cancer incidence, mortality, expected survival, and trends in incidence and mortality, and comorbidity information is missing [78,79]. If routinely collected health data can be linked to the regional mortality database and even to local cancer registries, future studies may assess the relationships of comorbidities, comorbid disease pairs, and their severities with different outcomes (eg, short-term and long-term mortality, and cancer-specific mortality).

### Conclusions

This study applied network analysis to routinely collected health data in order to examine the full spectrum of comorbidities and the complex coexisting relationships in newly diagnosed CRC patients. Some comorbidities, which were enriched in a given subgroup, largely increased the complexity of comorbid relationships, mediated correlations between other comorbidities, and might be the end points of many comorbidities. In addition, some co-occurring disease pairs predominated in a given subgroup. This study systematically analyzed the prevalence of individual diseases as well as the comorbid strength of coexisting diseases to provide a complete picture of comorbidities in CRC patients at diagnosis and also provide new insights into patterns of comorbidity. The data-driven discovery of comorbidity patterns may help improve the understanding of complex diseases, supplement traditional approaches in clinical studies, and help formulate appropriate preventive health measures to address high-risk comorbidities.

## Appendix

Multimedia Appendix 1 The frequency and prevalence of comorbidities in new colorectal cancer cases.

Multimedia Appendix 2 Comorbidity prevalence using a 3-year look-back period and its difference compared with using a 5-year look-back period.

Multimedia Appendix 3 Scatter plot of comorbidity prevalence using 3-year and 5-year look-back periods.

Multimedia Appendix 4 Comorbidity prevalence difference by sex using a 5-year look-back period.

Multimedia Appendix 5 Comorbidity prevalence difference by region using a 5-year look-back period.

Multimedia Appendix 6 Comorbidity prevalence difference by cancer site using a 5-year look-back period.

Multimedia Appendix 7 Prevalence differences of enrichment comorbidities in subgroups after adjustment with age.

## Abbreviations

CHF: congestive heart failure
COPD: chronic obstructive pulmonary disease
CRC: colorectal cancer
DM: diabetes mellitus
HDR: hospital discharge record
ICD-10: International Classification of Diseases, 10th Revision
IHD: ischemic heart disease
PCN: phenotypic comorbidity network

## Data Availability

The data sets generated or analyzed during this study are restricted by the Ethics Committee of the Health Information Center of Sichuan Province and are not publicly available.
