# Supplementary material for: Comorbidity Patterns in Patients Newly Diagnosed With Colorectal Cancer: Network-Based Study
Source: JMIR Public Health Surveill. 2023 Sep 5;9:e41999. doi: 10.2196/41999 (PMC10509734; doi:10.2196/41999)
Supplement: Multimedia Appendix 1 [file publichealth_v9i1e41999_app1.doc]

**Multimedia Appendix 1. The frequency and prevalence of comorbidities in new colorectal cancer cases.**

| ICD-10 | Disease name | Number of disease | Prevalence,% (95%CI) |
| --- | --- | --- | --- |
| I10 | Essential hypertension | 8581 | 29 (28.5,29.5)* |
| N40^a^ | Hyperplasia of prostate | 3816 | 21.9 (21.3,22.5)* |
| J44 | Other chronic obstructive pulmonary disease | 4199 | 14.2 (13.8,14.6)* |
| E11 | Diabetes mellitus | 3715 | 12.5 (12.2,12.9)* |
| I25 | Chronic ischaemic heart disease | 3200 | 10.8 (10.5,11.2)* |
| J43 | Emphysema | 2790 | 9.4 (9.1,9.8)* |
| E78 | Disorders of lipoprotein metabolism | 2718 | 9.2 (8.9,9.5)* |
| I70 | Atherosclerosis | 2735 | 9.2 (8.9,9.6)* |
| I63 | Cerebral infarction | 2641 | 8.9 (8.6,9.2)* |
| I50 | Heart failure | 2124 | 7.2 (6.9,7.5)* |
| I67 | Other cerebrovascular diseases | 1970 | 6.7 (6.4,6.9)* |
| I49 | Other cardiac arrhythmias | 1591 | 5.4 (5.1,5.6)* |
| J42 | Unspecified chronic bronchitis | 1469 | 5 (4.7,5.2)* |
| E46 | Unspecified protein-energy malnutrition | 1216 | 4.1 (3.9,4.3)* |
| K21 | Gastro-oesophageal reflux disease | 1128 | 3.8 (3.6,4)* |
| M47 | Spondylosis | 1126 | 3.8 (3.6,4)* |
| I51 | Complications and ill-defined descriptions of heart disease | 1100 | 3.7 (3.5,3.9)* |
| E77 | Disorders of glycoprotein metabolism | 1067 | 3.6 (3.4,3.8)* |
| G45 | Transient cerebral ischaemic attacks and related syndromes | 1019 | 3.4 (3.2,3.7)* |
| D86 | Sarcoidosis | 966 | 3.3 (3.1,3.5)* |
| G31 | Other degenerative diseases of nervous system | 907 | 3.1 (2.9,3.3)* |
| M81 | Osteoporosis without pathological fracture | 918 | 3.1 (2.9,3.3)* |
| I11 | Hypertensive heart disease | 897 | 3 (2.8,3.2)* |
| H25 | Senile cataract | 844 | 2.9 (2.7,3)* |
| I27 | Other pulmonary heart diseases | 828 | 2.8 (2.6,3)* |
| E14 | Unspecified diabetes mellitus | 795 | 2.7 (2.5,2.9)* |
| N19 | Unspecified renal failure | 742 | 2.5 (2.3,2.7)* |
| I48 | Atrial fibrillation and flutter | 706 | 2.4 (2.2,2.6)* |
| J47 | Bronchiectasis | 668 | 2.3 (2.1,2.4)* |
| E04 | Other nontoxic goitre | 638 | 2.2 (2,2.3)* |
| D68 | Other coagulation defects | 612 | 2.1 (1.9,2.2)* |
| K83 | Other diseases of biliary tract | 626 | 2.1 (2,2.3)* |
| I69 | Sequelae of cerebrovascular disease | 575 | 1.9 (1.8,2.1)* |
| I65 | Occlusion and stenosis of precerebral arteries | 572 | 1.9 (1.8,2.1)* |
| N18 | Chronic renal failure | 505 | 1.7 (1.6,1.9)* |
| I38 | Endocarditis, valve unspecified | 476 | 1.6 (1.5,1.8)* |
| K57 | Diverticular disease of intestine | 440 | 1.5 (1.4,1.6)* |
| K74 | Fibrosis and cirrhosis of liver | 453 | 1.5 (1.4,1.7)* |
| M17 | Gonarthrosis | 450 | 1.5 (1.4,1.7)* |
| J32 | Chronic sinusitis | 422 | 1.4 (1.3,1.6)* |
| E43 | Unspecified severe protein.energy malnutrition | 388 | 1.3 (1.2,1.4)* |
| I20 | Angina pectoris | 381 | 1.3 (1.2,1.4)* |
| I44 | Atrioventricular and left bundle-branch block | 385 | 1.3 (1.2,1.4)* |
| C34 | Malignant neoplasm of bronchus and lung | 364 | 1.2 (1.1,1.4)* |
| I45 | Other conduction disorders | 364 | 1.2 (1.1,1.4)* |
| M10 | Gout | 347 | 1.2 (1.1,1.3)* |
| C22 | Malignant neoplasm of liver and intrahepatic bile ducts | 338 | 1.1 (1.0,1.3)* |
| E27 | Other disorders of adrenal gland | 334 | 1.1 (1.0,1.3)* |
| C80 | Malignant neoplasm without specification of site | 329 | 1.1 (1.0,1.2)* |
| H26 | Other cataract | 311 | 1.1 (0.9,1.2) |
| D84 | Other immunodeficiencies | 302 | 1 (0.9,1.1) |
| C56^b^ | Malignant neoplasm of ovary | 111 | 0.9 (0.8,1.1) |
| C16 | Malignant neoplasm of stomach | 264 | 0.9 (0.8,1) |
| I35 | Nonrheumatic aortic valve disorders | 255 | 0.9 (0.8,1) |
| D89 | Other disorders involving the immune mechanism | 229 | 0.8 (0.7,0.9) |
| F41 | Other anxiety disorders | 233 | 0.8 (0.7,0.9) |
| I74 | Arterial embolism and thrombosis | 224 | 0.8 (0.7,0.9) |
| C61^a^ | Malignant neoplasm of prostate | 115 | 0.7 (0.5,0.8) |
| N32 | Other disorders of bladder | 217 | 0.7 (0.6,0.8) |
| D61 | Other aplastic anaemias | 187 | 0.6 (0.5,0.7) |
| I77 | Other disorders of arteries and arterioles | 164 | 0.6 (0.5,0.6) |
| I71 | Aortic aneurysm and dissection | 167 | 0.6 (0.5,0.7) |
| I47 | Paroxysmal tachycardia | 184 | 0.6 (0.5,0.7) |
| I08 | Multiple valve diseases | 190 | 0.6 (0.6,0.7) |
| N80^b^ | Endometriosis | 75 | 0.6 (0.5,0.8) |
| E72 | Other disorders of amino.acid metabolism | 161 | 0.5 (0.5,0.6) |
| * Prevalence was significantly greater than 1% (one-side test, *P* < .025); CI: confidence interval. a: male-specific disease; b: female-specific disease. | | | |
