# Supplementary material for: Comorbidity Patterns in Patients Newly Diagnosed With Colorectal Cancer: Network-Based Study
Source: JMIR Public Health Surveill. 2023 Sep 5;9:e41999. doi: 10.2196/41999 (PMC10509734; doi:10.2196/41999)
Supplement: Multimedia Appendix 2 [file publichealth_v9i1e41999_app2.doc]

**Multimedia Appendix 2. Comorbidity prevalence using a 3-year look-back period and its difference compared with using a 5-year look-back period.**

| ICD-10 | Prevalence, % (95%CI) | The absolute decrease in prevalence # | *P* |
| --- | --- | --- | --- |
| C16 | 0.9 (0.8, 1) | 0 | 0.2725 |
| C22 | 1.1 (1, 1.3)* | 0 | 0.4637 |
| C34 | 1.2 (1.1, 1.3)* | 0 | 0.285 |
| C56 | 0.9 (0.7, 1) | 0 | 0.258 |
| C61 | 0.6 (0.5, 0.8) | 0.1 | 0.2947 |
| C80 | 1.1 (1, 1.2) | 0 | 0.4632 |
| D61 | 0.6 (0.5, 0.6) | 0 | 0.0412 |
| D68 | 2 (1.9, 2.2)* | 0.1 | 0.3458 |
| D84 | 1 (0.9, 1.1) | 0 | 0.4386 |
| D86 | 3.2 (3, 3.4)* | 0.1 | 0.2695 |
| D89 | 0.8 (0.7, 0.9) | 0 | 0.4295 |
| E04 | 2 (1.9, 2.2)* | 0.2 | 0.0851 |
| E11 | 12.3 (11.9, 12.7)* | 0.2 | 0.091 |
| E14 | 2.3 (2.1, 2.5)* | 0.4 | <0.0001 |
| E27 | 1.1 (1, 1.2) | 0 | 0.3354 |
| E43 | 1.3 (1.2, 1.4)* | 0 | 0.3092 |
| E46 | 4 (3.8, 4.3)* | 0.1 | 0.2618 |
| E72 | 0.5 (0.4, 0.6) | 0 | 0.0436 |
| E77 | 3.3 (3.1, 3.5)* | 0.3 | 0.002 |
| E78 | 8 (7.7, 8.3)* | 1.2 | <0.0001 |
| F41 | 0.7 (0.6, 0.8) | 0.1 | 0.0066 |
| G31 | 2.7 (2.5, 2.9)* | 0.4 | 0.0001 |
| G45 | 2.7 (2.5, 2.9)* | 0.7 | <0.0001 |
| H25 | 2 (1.9, 2.2)* | 0.9 | <0.0001 |
| H26 | 0.7 (0.6, 0.8) | 0.4 | <0.0001 |
| I08 | 0.6 (0.5, 0.7) | 0 | 0.2607 |
| I10 | 28.1 (27.6, 28.6)* | 0.9 | 0.0005 |
| I11 | 2.6 (2.5, 2.8)* | 0.4 | <0.0001 |
| I20 | 1.1 (1, 1.2) | 0.2 | 0.0009 |
| I25 | 9.9 (9.6, 10.3)* | 0.9 | <0.0001 |
| I27 | 2.5 (2.3, 2.7)* | 0.3 | 0.0008 |
| I35 | 0.8 (0.7, 0.9) | 0.1 | 0.1906 |
| I38 | 1.5 (1.4, 1.6)* | 0.1 | 0.0454 |
| I44 | 1.2 (1.1, 1.3)* | 0.1 | 0.0979 |
| I45 | 1.1 (1, 1.3)* | 0.1 | 0.0671 |
| I47 | 0.6 (0.5, 0.6) | 0 | 0.0762 |
| I48 | 2.3 (2.1, 2.5)* | 0.1 | 0.1612 |
| I49 | 4.9 (4.7, 5.2)* | 0.5 | 0.0002 |
| I50 | 6.7 (6.4, 7)* | 0.5 | 0.0003 |
| I51 | 3.4 (3.2, 3.6)* | 0.3 | 0.0023 |
| I63 | 7.9 (7.6, 8.2)* | 1 | <0.0001 |
| I65 | 1.5 (1.4, 1.7)* | 0.4 | <0.0001 |
| I67 | 5.5 (5.3, 5.8)* | 1.2 | <0.0001 |
| I69 | 1.8 (1.7, 2)* | 0.1 | 0.0573 |
| I70 | 8.4 (8.1, 8.7)* | 0.8 | <0.0001 |
| I71 | 0.5 (0.5, 0.6) | 0.1 | 0.3281 |
| I74 | 0.7 (0.6, 0.8) | 0.1 | 0.2335 |
| I77 | 0.5 (0.4, 0.6) | 0.1 | 0.1351 |
| J32 | 1.2 (1, 1.3)* | 0.2 | <0.0001 |
| J42 | 4.5 (4.3, 4.8)* | 0.5 | 0.0001 |
| J43 | 9.1 (8.8, 9.4)* | 0.3 | 0.026 |
| J44 | 13.6 (13.2, 14)* | 0.6 | 0.0011 |
| J47 | 2.1 (1.9, 2.2)* | 0.2 | 0.019 |
| K21 | 3.7 (3.5, 3.9)* | 0.1 | 0.1116 |
| K57 | 1.5 (1.3, 1.6)* | 0 | 0.3376 |
| K74 | 1.5 (1.4, 1.7)* | 0 | 0.3756 |
| K83 | 2 (1.8, 2.2)* | 0.1 | 0.0768 |
| M10 | 1.1 (1, 1.2) | 0.1 | 0.0861 |
| M17 | 1.2 (1.1, 1.4)* | 0.3 | <0.0001 |
| M47 | 2.8 (2.7, 3)* | 1 | <0.0001 |
| M81 | 2.6 (2.4, 2.8)* | 0.5 | <0.0001 |
| N18 | 1.7 (1.5, 1.8)* | 0 | 0.2531 |
| N19 | 2.4 (2.2, 2.6)* | 0.1 | 0.1239 |
| N32 | 0.7 (0.6, 0.8) | 0 | 0.2089 |
| N40 | 20.6 (20, 21.2)* | 1.3 | <0.0001 |
| N80 | 0.5 (0.4, 0.7) | 0.1 | 0.0964 |
| CI: confidence interval; * prevalence was significantly greater than 1% (one-side test, *P* <.025); # The absolute decrease in prevalence compared with using a 5-year look-back period. | | | |
