# Supplementary material for: Comorbidity Patterns in Patients Newly Diagnosed With Colorectal Cancer: Network-Based Study
Source: JMIR Public Health Surveill. 2023 Sep 5;9:e41999. doi: 10.2196/41999 (PMC10509734; doi:10.2196/41999)
Supplement: Multimedia Appendix 3 [file publichealth_v9i1e41999_app3.doc]

**Multimedia Appendix 3. Scatter plot of comorbidity prevalence using 3-year and 5-year look-back periods.**


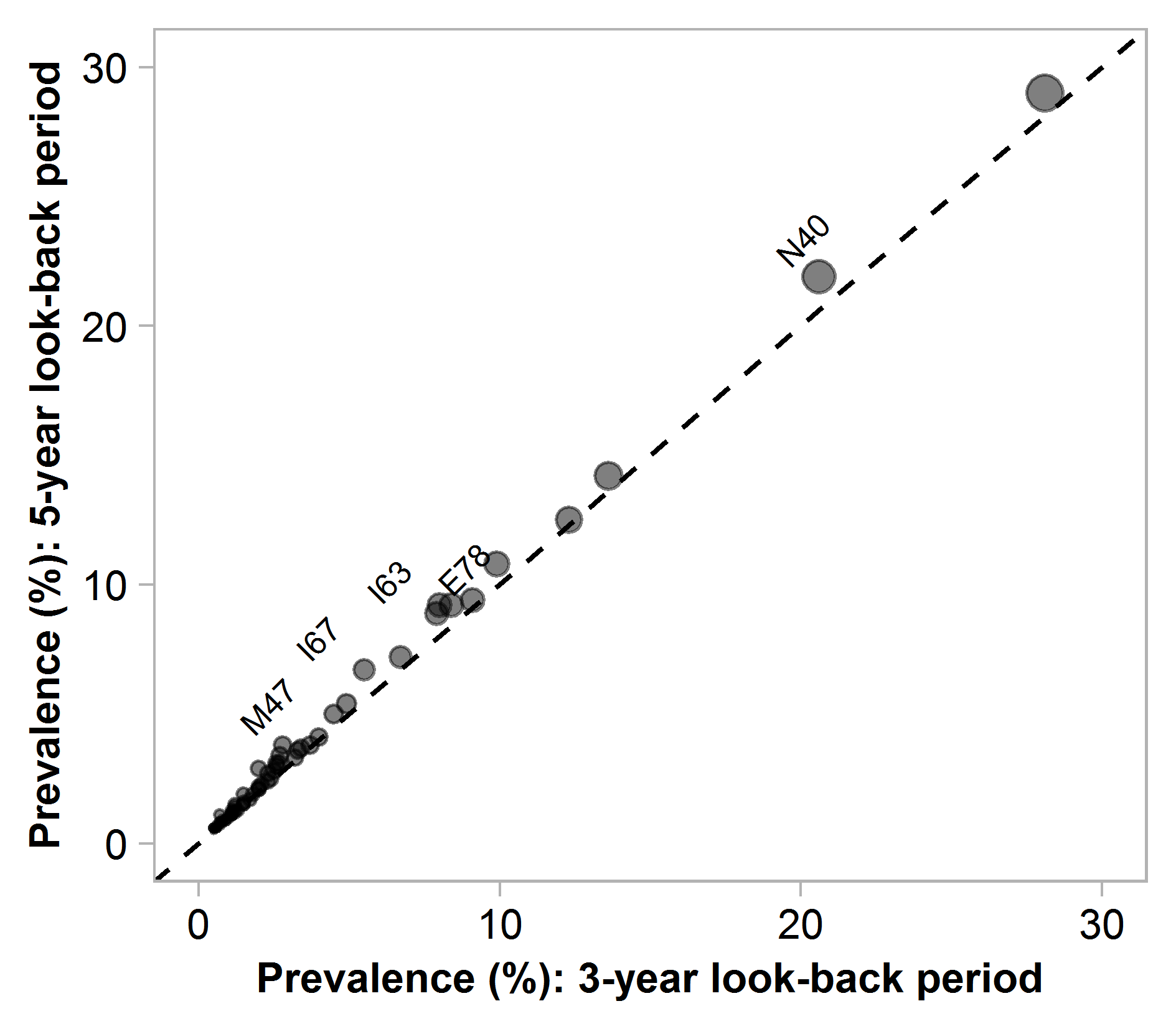


Comorbidity with the absolute decrease prevalence > 1% compared with using a 5-year look-back period is labeled with ICD-10. N40: hyperplasia of prostate, E78: disorders of lipoprotein metabolism, I67: other cerebrovascular diseases, I63: cerebral infarction, M47: spondylosis.
