# Supplementary material for: Comorbidity Patterns in Patients Newly Diagnosed With Colorectal Cancer: Network-Based Study
Source: JMIR Public Health Surveill. 2023 Sep 5;9:e41999. doi: 10.2196/41999 (PMC10509734; doi:10.2196/41999)
Supplement: Multimedia Appendix 4 [file publichealth_v9i1e41999_app4.doc]

**Multimedia Appendix 4. Comorbidity prevalence difference by sex using a 5-year look-back period.**

| ICD-10 | Prevalence by sex,% (95%CI) | | Absolute difference M-F, % (95%CI) | Relative difference | Significant difference# |
| --- | --- | --- | --- | --- | --- |
|  | Females | Males |  |  |  |
| C22 | 0.9 (0.7,1.1) | 1.3 (1.1,1.5)* | 0.4 (0.2,0.6) | 0.4 | higher in males |
| C34 | 1 (0.8,1.1) | 1.4 (1.2,1.6)* | 0.5 (0.2,0.7) | 0.4 | higher in males |
| D68 | 1.9 (1.7,2.2)* | 2.2 (2,2.4)* | 0.2 (-0.1,0.6) | 0.1 | unsignificant |
| D86 | 3.2 (2.8,3.5)* | 3.3 (3.1,3.6)* | 0.2 (-0.2,0.6) | 0.1 | unsignificant |
| E04 | 3.3 (3,3.6)* | 1.3 (1.2,1.5)* | -2 (-2.3,-1.6) | -0.8 | higher in females |
| E11 | 13.3 (12.7,13.9)* | 12.1 (11.6,12.5)* | -1.2 (-2,-0.4) | -0.1 | unsignificant |
| E14 | 2.6 (2.4,2.9)* | 2.7 (2.5,3)* | 0.1 (-0.3,0.4) | 0 | unsignificant |
| E27 | 1.2 (1,1.4)* | 1.1 (0.9,1.2) | -0.1 (-0.4,0.1) | -0.1 | unsignificant |
| E43 | 1.4 (1.2,1.6)* | 1.3 (1.1,1.5)* | -0.1 (-0.4,0.2) | -0.1 | unsignificant |
| E46 | 4.1 (3.8,4.5)* | 4.1 (3.8,4.4)* | -0.1 (-0.5,0.4) | 0 | unsignificant |
| E77 | 3.3 (3,3.7)* | 3.8 (3.5,4.1)* | 0.4 (0,0.9) | 0.1 | unsignificant |
| E78 | 10.8 (10.2,11.3)* | 8.1 (7.7,8.5)* | -2.7 (-3.4,-2) | -0.3 | higher in females |
| F41 | 1.3 (1.1,1.5)* | 0.4 (0.3,0.6) | -0.8 (-1.1,-0.6) | -1 | higher in females |
| G31 | 2.6 (2.3,2.9)* | 3.4 (3.1,3.7)* | 0.8 (0.4,1.1) | 0.3 | higher in males |
| G45 | 3.6 (3.3,4)* | 3.3 (3.1,3.6)* | -0.3 (-0.7,0.1) | -0.1 | unsignificant |
| H25 | 3.3 (3,3.7)* | 2.5 (2.3,2.8)* | -0.8 (-1.2,-0.4) | -0.3 | higher in females |
| H26 | 1.2 (1,1.4)* | 0.9 (0.8,1.1) | -0.3 (-0.5,0) | -0.2 | unsignificant |
| I10 | 29.7 (28.9,30.5)* | 28.5 (27.8,29.2)* | -1.2 (-2.3,-0.1) | 0 | unsignificant |
| I11 | 3 (2.7,3.3)* | 3.1 (2.8,3.3)* | 0.1 (-0.3,0.5) | 0 | unsignificant |
| I20 | 1 (0.9,1.2) | 1.5 (1.3,1.7)* | 0.4 (0.2,0.7) | 0.4 | higher in males |
| I25 | 11.9 (11.3,12.4)* | 10.1 (9.6,10.5)* | -1.8 (-2.5,-1.1) | -0.2 | higher in females |
| I27 | 2.2 (1.9,2.5)* | 3.2 (3,3.5)* | 1 (0.6,1.4) | 0.4 | higher in males |
| I38 | 1.8 (1.6,2.1)* | 1.4 (1.3,1.6)* | -0.4 (-0.7,-0.1) | -0.2 | higher in females |
| I44 | 0.9 (0.7,1) | 1.6 (1.4,1.8)* | 0.8 (0.5,1) | 0.6 | higher in males |
| I45 | 0.8 (0.7,1) | 1.5 (1.3,1.7)* | 0.7 (0.5,1) | 0.6 | higher in males |
| I48 | 2.5 (2.2,2.8)* | 2.3 (2.1,2.6)* | -0.2 (-0.5,0.2) | -0.1 | unsignificant |
| I49 | 5.4 (5,5.8)* | 5.4 (5.1,5.7)* | 0 (-0.5,0.5) | 0 | unsignificant |
| I50 | 7.3 (6.8,7.7)* | 7.1 (6.7,7.5)* | -0.2 (-0.8,0.4) | 0 | unsignificant |
| I51 | 4.2 (3.8,4.5)* | 3.4 (3.1,3.7)* | -0.7 (-1.2,-0.3) | -0.2 | higher in females |
| I63 | 8.6 (8.1,9.1)* | 9.1 (8.7,9.6)* | 0.6 (-0.1,1.2) | 0.1 | unsignificant |
| I65 | 1.7 (1.4,1.9)* | 2.1 (1.9,2.3)* | 0.5 (0.2,0.8) | 0.2 | higher in males |
| I67 | 7.1 (6.6,7.6)* | 6.3 (6,6.7)* | -0.7 (-1.3,-0.2) | -0.1 | unsignificant |
| I69 | 1.6 (1.3,1.8)* | 2.2 (2,2.4)* | 0.6 (0.3,1) | 0.3 | higher in males |
| I70 | 8.9 (8.4,9.5)* | 9.5 (9,9.9)* | 0.5 (-0.1,1.2) | I70 | unsignificant |
| J32 | 1.3 (1.1,1.5)* | 1.5 (1.3,1.7)* | 0.2 (-0.1,0.5) | J32 | unsignificant |
| J42 | 4 (3.7,4.4)* | 5.6 (5.3,6)* | 1.6 (1.1,2.1) | J42 | higher in males |
| J43 | 5.4 (5,5.8)* | 12.3 (11.8,12.8)* | 6.9 (6.3,7.5) | J43 | higher in males |
| J44 | 9.5 (9,10)* | 17.4 (16.9,18)* | 7.9 (7.2,8.7) | J44 | higher in males |
| J47 | 2 (1.8,2.3)* | 2.4 (2.2,2.7)* | 0.4 (0.1,0.8) | J47 | higher in males |
| K21 | 4 (3.6,4.3)* | 3.7 (3.4,4)* | -0.3 (-0.7,0.2) | K21 | unsignificant |
| K57 | 1.5 (1.3,1.7)* | 1.5 (1.3,1.7)* | 0 (-0.3,0.3) | K57 | unsignificant |
| K74 | 1.2 (1.1,1.5)* | 1.7 (1.5,1.9)* | 0.5 (0.2,0.8) | K74 | higher in males |
| K83 | 2.4 (2.1,2.7)* | 1.9 (1.7,2.1)* | -0.5 (-0.8,-0.1) | K83 | higher in females |
| M10 | 0.3 (0.2,0.4) | 1.8 (1.6,2)* | 1.5 (1.3,1.7) | M10 | higher in males |
| M17 | 2.3 (2,2.6)* | 1 (0.9,1.2) | -1.3 (-1.6,-1) | M17 | higher in females |
| M47 | 4.4 (4.1,4.8)* | 3.4 (3.1,3.6)* | -1.1 (-1.5,-0.6) | M47 | higher in females |
| M81 | 5.1 (4.8,5.6)* | 1.7 (1.5,1.9)* | -3.5 (-3.9,-3) | M81 | higher in females |
| N18 | 1.3 (1.1,1.5)* | 2 (1.8,2.2)* | 0.7 (0.4,1) | N18 | higher in males |
| N19 | 1.8 (1.6,2)* | 3 (2.8,3.3)* | 1.2 (0.9,1.6) | N19 | higher in males |
| CI: confidence interval; * prevalence was significantly greater than 1% (one-side test, *P* < .025); Absolute difference=Male-Female; Relative difference=2(Male-Female)/(Male+Female); # if absolute difference was statistically significance after Bonferroni correction and relative difference > 0.1, then difference by sex was significant. | | | | | |
