# Supplementary material for: Comorbidity Patterns in Patients Newly Diagnosed With Colorectal Cancer: Network-Based Study
Source: JMIR Public Health Surveill. 2023 Sep 5;9:e41999. doi: 10.2196/41999 (PMC10509734; doi:10.2196/41999)
Supplement: Multimedia Appendix 5 [file publichealth_v9i1e41999_app5.doc]

**Multimedia Appendix 5. Comorbidity prevalence difference by region using a 5-year look-back period.**

| ICD-10 | Prevalence by region, % (95%CI) | | Absolute difference , % (95%CI) | Relative difference | Significant difference# |
| --- | --- | --- | --- | --- | --- |
|  | rural | urban |  |  |  |
| C22 | 1.2 (1.1,1.4)* | 1.1 (0.9,1.2) | -0.2 (-0.4,0.1) | -0.1 | unsignificant |
| C34 | 1.2 (1,1.3)* | 1.3 (1.1,1.5)* | 0.1 (-0.1,0.4) | 0.1 | unsignificant |
| D68 | 1.8 (1.6,2)* | 2.4 (2.2,2.7)* | 0.6 (0.3,1) | 0.3 | higher in urban |
| D86 | 3.1 (2.8,3.4)* | 3.5 (3.2,3.8)* | 0.4 (0,0.8) | 0.1 | unsignificant |
| E04 | 1.7 (1.5,1.9)* | 2.7 (2.5,3)* | 1.1 (0.7,1.4) | 0.5 | higher in urban |
| E11 | 11.1 (10.6,11.6)* | 14.2 (13.7,14.8)* | 3.1 (2.4,3.9) | 0.2 | higher in urban |
| E14 | 2.5 (2.3,2.8)* | 2.9 (2.6,3.2)* | 0.4 (0,0.7) | 0.1 | unsignificant |
| E27 | 0.8 (0.7,1) | 1.5 (1.3,1.7)* | 0.7 (0.5,0.9) | 0.6 | higher in urban |
| E43 | 1.1 (1,1.3) | 1.6 (1.4,1.8)* | 0.4 (0.2,0.7) | 0.3 | higher in urban |
| E46 | 3.8 (3.5,4.1)* | 4.5 (4.1,4.8)* | 0.7 (0.3,1.2) | 0.2 | higher in urban |
| E77 | 3.5 (3.2,3.8)* | 3.7 (3.4,4.1)* | 0.2 (-0.2,0.7) | 0.1 | unsignificant |
| E78 | 7.9 (7.5,8.3)* | 10.7 (10.2,11.2)* | 2.8 (2.1,3.5) | 0.3 | higher in urban |
| G31 | 2.8 (2.5,3)* | 3.4 (3.1,3.7)* | 0.6 (0.2,1) | 0.2 | higher in urban |
| G45 | 2.9 (2.6,3.1)* | 4.1 (3.8,4.4)* | 1.2 (0.8,1.7) | 0.4 | higher in urban |
| H25 | 2.6 (2.4,2.9)* | 3.1 (2.8,3.4)* | 0.5 (0.1,0.8) | 0.2 | unsignificant |
| H26 | 0.9 (0.8,1.1) | 1.2 (1,1.4)* | 0.3 (0,0.5) | 0.2 | unsignificant |
| I10 | 27.4 (26.7,28.1)* | 30.9 (30.1,31.6)* | 3.5 (2.4,4.5) | 0.1 | unsignificant |
| I11 | 2.3 (2.1,2.5)* | 3.9 (3.6,4.3)* | 1.6 (1.2,2) | 0.5 | higher in urban |
| I20 | 1 (0.8,1.1) | 1.7 (1.5,1.9)* | 0.7 (0.5,1) | 0.6 | higher in urban |
| I25 | 10.6 (10.1,11)* | 11.1 (10.6,11.7)* | 0.6 (-0.1,1.3) | 0.1 | unsignificant |
| I27 | 3 (2.8,3.3)* | 2.5 (2.3,2.8)* | -0.5 (-0.9,-0.1) | -0.2 | higher in rural |
| I38 | 1.5 (1.3,1.7)* | 1.7 (1.5,1.9)* | 0.2 (-0.1,0.5) | 0.1 | unsignificant |
| I44 | 1.1 (0.9,1.2) | 1.6 (1.4,1.8)* | 0.5 (0.2,0.8) | 0.4 | higher in urban |
| I45 | 1 (0.9,1.2) | 1.5 (1.3,1.7)* | 0.5 (0.2,0.7) | 0.4 | higher in urban |
| I48 | 2.1 (1.9,2.4)* | 2.7 (2.4,3)* | 0.5 (0.2,0.9) | 0.2 | higher in urban |
| I49 | 4.7 (4.3,5)* | 6.2 (5.8,6.6)* | 1.5 (1,2.1) | 0.3 | higher in urban |
| I50 | 7 (6.7,7.5)* | 7.4 (6.9,7.8)* | 0.3 (-0.3,0.9) | 0 | unsignificant |
| I51 | 3.7 (3.4,4)* | 3.8 (3.5,4.1)* | 0.1 (-0.3,0.6) | 0 | unsignificant |
| I63 | 7.3 (6.9,7.8)* | 10.8 (10.3,11.3)* | 3.5 (2.8,4.1) | 0.4 | higher in urban |
| I65 | 1.4 (1.2,1.6)* | 2.5 (2.3,2.8)* | 1.1 (0.8,1.4) | 0.6 | higher in urban |
| I67 | 5 (4.7,5.4)* | 8.6 (8.1,9.1)* | 3.6 (3,4.2) | 0.5 | higher in urban |
| I69 | 1.6 (1.4,1.8)* | 2.3 (2,2.6)* | 0.7 (0.3,1) | 0.3 | higher in urban |
| I70 | 7.3 (6.9,7.7)* | 11.5 (11,12.1)* | 4.2 (3.5,4.9) | 0.4 | higher in urban |
| J32 | 1.3 (1.1,1.5)* | 1.6 (1.4,1.8)* | 0.2 (0,0.5) | 0.2 | unsignificant |
| J42 | 5.3 (4.9,5.6)* | 4.6 (4.3,5)* | -0.6 (-1.1,-0.1) | -0.1 | unsignificant |
| J43 | 9 (8.6,9.5)* | 9.9 (9.4,10.4)* | 0.9 (0.2,1.6) | 0.1 | unsignificant |
| J44 | 14.4 (13.9,15)* | 13.8 (13.2,14.4)* | -0.6 (-1.4,0.2) | 0 | unsignificant |
| J47 | 2.1 (1.9,2.3)* | 2.5 (2.2,2.8)* | 0.4 (0.1,0.7) | 0.2 | unsignificant |
| K21 | 2.7 (2.5,3)* | 5.1 (4.7,5.4)* | 2.3 (1.9,2.8) | 0.6 | higher in urban |
| K57 | 1.2 (1,1.4)* | 1.8 (1.6,2.1)* | 0.6 (0.3,0.9) | 0.4 | higher in urban |
| K74 | 1.5 (1.3,1.7)* | 1.6 (1.4,1.8)* | 0.1 (-0.2,0.4) | 0.1 | unsignificant |
| K83 | 2 (1.8,2.2)* | 2.2 (2,2.5)* | 0.2 (-0.1,0.6) | 0.1 | unsignificant |
| M10 | 0.9 (0.8,1.1) | 1.5 (1.3,1.7)* | 0.6 (0.3,0.8) | 0.5 | higher in urban |
| M17 | 1.2 (1,1.3) | 2 (1.7,2.2)* | 0.8 (0.5,1.1) | 0.5 | higher in urban |
| M47 | 3 (2.8,3.3)* | 4.7 (4.3,5.1)* | 1.7 (1.2,2.1) | 0.4 | higher in urban |
| M81 | 2.5 (2.3,2.8)* | 3.8 (3.5,4.1)* | 1.3 (0.9,1.7) | 0.4 | higher in urban |
| N18 | 1.2 (1,1.4)* | 2.3 (2.1,2.6)* | 1.1 (0.8,1.4) | 0.6 | higher in urban |
| N19 | 2.5 (2.2,2.7)* | 2.5 (2.3,2.8)* | 0.1 (-0.3,0.4) | 0 | unsignificant |
| N40 | 20.1 (19.3,20.9)* | 24.1 (23.1,25)* | 3.9 (2.7,5.2) | 0.2 | higher in urban |
| CI: confidence interval; * prevalence was significantly greater than 1% (one-side test, *P* < .025); Absolute difference=urban-rural; Relative difference=2(urban-rural)/(urban+rural); # if absolute difference was statistically significance after Bonferroni correction and relative difference >0.1, then difference by region was significant. | | | | | |
