# Supplementary material for: Comorbidity Patterns in Patients Newly Diagnosed With Colorectal Cancer: Network-Based Study
Source: JMIR Public Health Surveill. 2023 Sep 5;9:e41999. doi: 10.2196/41999 (PMC10509734; doi:10.2196/41999)
Supplement: Multimedia Appendix 6 [file publichealth_v9i1e41999_app6.doc]

**Multimedia Appendix 6. Comorbidity prevalence difference by cancer site using a 5-year look-back period.**

| ICD-10 | Prevalence by cancer site,% (95%CI) | | Absolute difference , % (95%CI) | Relative difference | Significant difference# |
| --- | --- | --- | --- | --- | --- |
|  | rectal | colon |  |  |  |
| C22 | 0.9 (0.8,1.1) | 1.4 (1.2,1.7)* | 0.5 (0.2,0.7) | 0.4 | higher in colon |
| C34 | 1.1 (1,1.3) | 1.3 (1.1,1.6)* | 0.2 (0,0.5) | 0.2 | unsignificant |
| C80 | 0.9 (0.8,1.1) | 1.4 (1.2,1.6)* | 0.4 (0.2,0.7) | 0.4 | higher in colon |
| D68 | 1.6 (1.4,1.8)* | 2.7 (2.4,3)* | 1 (0.7,1.4) | 0.5 | higher in colon |
| D86 | 3.4 (3.1,3.7)* | 3.1 (2.9,3.5)* | -0.2 (-0.6,0.2) | -0.1 | unsignificant |
| E04 | 1.9 (1.7,2.1)* | 2.5 (2.2,2.7)* | 0.6 (0.2,0.9) | 0.3 | higher in colon |
| E11 | 11.8 (11.3,12.3)* | 13.6 (13,14.2)* | 1.8 (1,2.6) | 0.1 | unsignificant |
| E14 | 2.5 (2.3,2.8)* | 2.9 (2.6,3.2)* | 0.4 (0,0.8) | 0.2 | unsignificant |
| E27 | 1 (0.9,1.2) | 1.3 (1.1,1.5)* | 0.3 (0,0.5) | 0.2 | unsignificant |
| E43 | 1.1 (0.9,1.3) | 1.6 (1.4,1.9)* | 0.5 (0.2,0.8) | 0.4 | higher in colon |
| E46 | 3.5 (3.2,3.8)* | 4.9 (4.5,5.3)* | 1.4 (0.9,1.9) | 0.3 | higher in colon |
| E77 | 2.8 (2.6,3.1)* | 4.6 (4.3,5)* | 1.8 (1.3,2.2) | 0.5 | higher in colon |
| E78 | 8.7 (8.3,9.2)* | 9.6 (9.1,10.1)* | 0.8 (0.2,1.5) | 0.1 | unsignificant |
| G31 | 2.9 (2.6,3.1)* | 3.3 (3,3.6)* | 0.4 (0,0.8) | 0.1 | unsignificant |
| G45 | 3.3 (3,3.6)* | 3.7 (3.3,4)* | 0.4 (0,0.8) | 0.1 | unsignificant |
| H25 | 2.8 (2.5,3)* | 3 (2.7,3.3)* | 0.2 (-0.2,0.6) | 0.1 | unsignificant |
| I10 | 27.9 (27.2,28.6)* | 30.5 (29.7,31.3)* | 2.5 (1.5,3.6) | 0.1 | unsignificant |
| I11 | 2.7 (2.5,3)* | 3.5 (3.2,3.8)* | 0.7 (0.3,1.1) | 0.2 | higher in colon |
| I20 | 1 (0.9,1.2) | 1.6 (1.4,1.9)* | 0.6 (0.4,0.9) | 0.5 | higher in colon |
| I25 | 9.6 (9.1,10)* | 12.4 (11.9,13)* | 2.9 (2.1,3.6) | 0.3 | higher in colon |
| I27 | 2.8 (2.5,3.1)* | 2.8 (2.5,3.1)* | 0 (-0.4,0.4) | 0 | unsignificant |
| I38 | 1.7 (1.5,1.9)* | 1.6 (1.4,1.8)* | -0.1 (-0.4,0.2) | -0.1 | unsignificant |
| I44 | 1.3 (1.1,1.5)* | 1.4 (1.2,1.6)* | 0.1 (-0.2,0.4) | 0.1 | unsignificant |
| I45 | 1.2 (1,1.4)* | 1.3 (1.1,1.5)* | 0.1 (-0.2,0.3) | 0 | unsignificant |
| I48 | 2.2 (2,2.4)* | 2.7 (2.5,3)* | 0.6 (0.2,0.9) | 0.2 | higher in colon |
| I49 | 5.2 (4.9,5.6)* | 5.7 (5.3,6.1)* | 0.5 (-0.1,1) | 0.1 | unsignificant |
| I50 | 6.2 (5.8,6.6)* | 8.5 (8.1,9)* | 2.3 (1.7,3) | 0.3 | higher in colon |
| I51 | 3.4 (3.1,3.7)* | 4.2 (3.9,4.6)* | 0.8 (0.4,1.3) | 0.2 | higher in colon |
| I63 | 8.1 (7.7,8.5)* | 10 (9.5,10.5)* | 1.9 (1.2,2.6) | 0.2 | higher in colon |
| I65 | 1.8 (1.6,2.1)* | 2.1 (1.9,2.3)* | 0.2 (-0.1,0.6) | 0.1 | unsignificant |
| I67 | 6 (5.6,6.3)* | 7.4 (7,7.9)* | 1.5 (0.9,2.1) | 0.2 | higher in colon |
| I69 | 1.7 (1.5,1.9)* | 2.2 (2,2.5)* | 0.5 (0.2,0.8) | 0.3 | higher in colon |
| I70 | 8.6 (8.1,9)* | 10.2 (9.7,10.7)* | 1.6 (0.9,2.3) | 0.2 | higher in colon |
| J32 | 1.4 (1.2,1.6)* | 1.3 (1.1,1.5)* | -0.1 (-0.3,0.2) | -0.1 | unsignificant |
| J42 | 4.9 (4.5,5.2)* | 5.1 (4.8,5.5)* | 0.3 (-0.2,0.8) | 0.1 | unsignificant |
| J43 | 9.9 (9.5,10.4)* | 9 (8.6,9.5)* | -0.9 (-1.6,-0.2) | -0.1 | unsignificant |
| J44 | 14.2 (13.7,14.8)* | 14.3 (13.7,14.9)* | 0 (-0.8,0.8) | 0 | unsignificant |
| J47 | 2.2 (2,2.4)* | 2.4 (2.1,2.6)* | 0.2 (-0.2,0.5) | 0.1 | unsignificant |
| K21 | 3.1 (2.9,3.4)* | 4.7 (4.3,5.1)* | 1.6 (1.1,2) | 0.4 | higher in colon |
| K57 | 1.3 (1.1,1.5)* | 1.7 (1.5,2)* | 0.4 (0.2,0.7) | 0.3 | higher in colon |
| K74 | 1.3 (1.1,1.5)* | 1.9 (1.6,2.1)* | 0.6 (0.3,0.9) | 0.4 | higher in colon |
| K83 | 1.7 (1.5,1.9)* | 2.6 (2.4,2.9)* | 0.9 (0.6,1.3) | 0.4 | higher in colon |
| M10 | 1.1 (1,1.3) | 1.2 (1,1.4)* | 0 (-0.2,0.3) | 0 | unsignificant |
| M17 | 1.4 (1.2,1.6)* | 1.6 (1.4,1.9)* | 0.2 (-0.1,0.5) | 0.1 | unsignificant |
| M47 | 3.4 (3.1,3.7)* | 4.3 (4,4.7)* | 0.9 (0.5,1.4) | 0.2 | higher in colon |
| M81 | 2.7 (2.5,3)* | 3.4 (3.1,3.8)* | 0.7 (0.3,1.1) | 0.2 | higher in colon |
| N18 | 1.5 (1.3,1.7)* | 2 (1.8,2.3)* | 0.5 (0.2,0.8) | 0.3 | higher in colon |
| N19 | 2.2 (2,2.5)* | 2.9 (2.6,3.2)* | 0.7 (0.3,1.1) | 0.3 | higher in colon |
| N40 | 21.5 (20.7,22.3)* | 22.6 (21.6,23.6)* | 1.1 (-0.2,2.3) | 0 | unsignificant |
| CI: confidence interval; * prevalence was significantly greater than 1% (one-side test, *P* < .025); Absolute difference=colon-rectal; Relative difference=2(colon-rectal)/(colon+rectal); # if absolute difference was statistically significance after Bonferroni correction and relative difference >0.1, then difference by cancer site was significant. | | | | | |
