# Supplementary material for: Comorbidity Patterns in Patients Newly Diagnosed With Colorectal Cancer: Network-Based Study
Source: JMIR Public Health Surveill. 2023 Sep 5;9:e41999. doi: 10.2196/41999 (PMC10509734; doi:10.2196/41999)
Supplement: Multimedia Appendix 7 [file publichealth_v9i1e41999_app7.doc]

**Multimedia Appendix 7.** **Prevalence differences of enrichment comorbidities in subgroups after adjustment with age.**

| ICD-10 | Enrichment comorbidities | Crude prevalence (95%CI) | prevalence difference after  adjustment with age (95% CI)# | | |
| --- | --- | --- | --- | --- | --- |
|  |  |  | Male-Female | Urban-Rural | Colon-Rectal |
| C22 | Malignant neoplasm of liver and intrahepatic bile ducts | 1.1 (1.0,1.3) | - | - | 0.5 (0.3, 0.7) |
| D68 | Other coagulation defects | 2.1 (1.9, 2.2) | - | - | 1.0 (0.7, 1.2) |
| E04 | Other nontoxic goitre | 2.2 (2.0, 2.3) | -2.0 (-2.2, -1.8) | 1.1 (0.8, 1.3) | - |
| E27 | Other disorders of adrenal gland | 1.1 (1.0, 1.3) | - | 0.7 (0.5, 0.9) | - |
| E77 | Disorders of glycoprotein metabolism | 3.6 (3.4, 3.8) | - | - | 1.7 (1.4, 2.0) |
| F41 | Other anxiety disorders | 0.8 (0.7, 0.9) | -0.8 (-1.0, -0.7) | - | - |
| I11 | Hypertensive heart disease | 3.0 (2.8, 3.2) | - | 1.5 (1.3, 1.8) |  |
| I20 | Angina pectoris | 1.3 (1.2, 1.4) | - | 0.7 (0.5, 0.9) | 0.6 (0.4, 0.8) |
| I44 | Atrioventricular and left bundle-branch block | 1.3 (1.2, 1.4) | 0.8 (0.6, 0.9) | - | - |
| I45 | Other conduction disorders | 1.2 (1.1, 1.4) | 0.7 (0.5, 0.9) | - | - |
| I65 | Occlusion and stenosis of precerebral arteries | 1.9 (1.8, 2.1) | - | 1.1 (0.9, 1.3) | - |
| I67 | Other cerebrovascular diseases | 6.7 (6.4, 6.9) | - | 3.4 (3.0, 3.8) | - |
| I70 | Atherosclerosis | 9.2 (8.9, 9.6) | - | 4 (3.5, 4.5) | - |
| J43 | Emphysema | 9.4 (9.1, 9.8) | 6.8 (6.3, 7.2) | - | - |
| J44 | COPD | 14.2 (13.8, 14.6) | 7.8 (7.3, 8.3) | - | - |
| K21 | Gastro-oesophageal reflux disease | 3.8 (3.6, 4.0) | - | 2.3 (2.0, 2.6) | 1.6 (1.3, 1.9) |
| K57 | Diverticular disease of intestine | 1.5 (1.4,1.6) | - | 0.6 (0.4, 0.8) | - |
| K83 | Other diseases of biliary tract | 2.1 (2.0,2.3) | - | - | 0.9 (0.7, 1.1) |
| M10 | Gout | 1.2 (1.1, 1.3) | 1.5 (1.4, 1.7) | 0.5 (0.4, 0.7) | - |
| M17 | Gonarthrosis | 1.5 (1.4, 1.7) | -1.3 (-1.5, -1.1) | 0.8 (0.6, 1.0) | - |
| M47 | Spondylosis | 3.8 (3.6, 4.0) | - | 1.6 (1.3, 1.9) | - |
| M81 | Osteoporosis without pathological fracture | 3.1 (2.9, 3.3) | -3.5 (-3.8, -3.2) | - | - |
| N18 | Chronic renal failure | 1.7 (1.6, 1.9) | 0.7 (0.5, 0.9) | 1.0 (0.8, 1.2) | - |
| N19 | Unspecified renal failure | 2.5 (2.3, 2.7) | 1.3 (1.0, 1.5) | - | - |
| CI: confidence interval. COPD: chronic obstructive pulmonary disease. #: the absolute prevalence differences by sex, region and cancer site were statistically significant after Bonferroni correction. -: the comorbidity was not enrichment in this subgroup. | | | | | |
